# Mixed methods investigation into provision of family rooms and support for parents admitted to psychiatric inpatient units and their children: Study protocol

**DOI:** 10.1371/journal.pone.0355693

**Published:** 2026-09-15

**Authors:** Abby Dunn, Chloe Elsby-Pearson, Luise Kalus, Mark Oliver, Eleanor Ratcliffe, Sam Cartwright-Hatton

**Affiliations:** 1 Department of Psychology, University of Sussex, Brighton, East Sussex, United Kingdom; 2 Sussex Partnership NHS Foundation Trust, Worthing, East Sussex, United Kingdom; 3 Expert by Experience Co-Applicant, Chichester, West Sussex, United Kingdom; 4 University of Surrey, Guildford, Surrey, United Kingdom; PLOS: Public Library of Science, UNITED KINGDOM OF GREAT BRITAIN AND NORTHERN IRELAND

## Abstract

**Background:**

Of 97,000 psychiatric inpatient admissions in England in 2022, 12–45% are thought to have had dependent children. The hospitalisation of parents requires separation from children; this is a source of distress for both parents and their children and can exacerbate parental symptoms, disrupt the parent-child relationship and is associated with negative child outcomes. Currently, most psychiatric wards have inadequate provision for child visitation, including unsatisfactory family rooms. Improved provision for family visits has been identified as a mechanism through which parent-child connection could be improved (1,2). This study aims to capture the perception and experience of family visitations during parents’ psychiatric hospitalisation from the perspective of parents, carers, children and young people (CYP) and healthcare professionals (HCP).

**Methods:**

The study employs an exploratory sequential mixed method design in which qualitative data collected during online interviews (work package 1) will inform the design of a subsequent questionnaire (work package 2). The results of both will be utilised to inform the co-production process for a future Family Visit Pack. Participants (12–16 parents, 6–10 carers, 12–16 HCP) will partake in 0–60-minute semi-structured interviews. In additional 6–10 CYPs will partake in interviews, using the “draw, write, tell” approach to share their experiences. The data from these interviews will be transcribed, anonymised and analysed with a five-stage framework analysis. Results from the interviews will inform the questionnaires for work package 2 which 50 parents, 25 HCPs and 25 carers will complete. Qualitative data will be subject to framework analysis and descriptive statistics will be generated from the questionnaire data. Development of topic guides, interview administration, framework analysis and questionnaire development will be supported by lived experience researchers.

**Discussion:**

This research will allow a better understanding of the experiences of families when visiting parents in inpatient units, as well as exploring which environmental and service delivery changes are priorities for parents, CYP, carers and HCPs.

**Trial Registrations:**

IRAS Number: IRAS 340906. ISRCTN – The UK’s Clinical Study Registry: ISRCTN69583343, registered 24/02/2026. https://doi.org/10.1186/ISRCTN69583343

## Introduction

Of 97,000 psychiatric inpatient admissions in England in 2022, 12–45% are thought to have had dependent children [1–3]. Hospitalisation is reserved only for patients with the highest mental health need (most frequently schizophrenia or highest-risk mood disorders). The hospitalisation of parents requires separation from children which is a source of distress to parent and child and can exacerbate parental symptoms, disrupt the parent-child relationship and is associated with impaired child outcomes including elevated risk of psychological difficulties [4–6]. Furthermore, hospitalisation often follows a period of acute ill-health (over one-third of admissions are compulsory detentions), which, coupled with high levels of readmission, can amplify strain on the family relationship [7]. Supporting the parent-child relationship during hospitalisation would contribute to minimising these risks.

Currently, most psychiatric wards have inadequate provision for child visitation. A 2007 Barnardo’s report and a 2019 Scottish audit highlighted inadequate provision for child visitation, including unsatisfactory family rooms [8]. In patient and public involvement work by the present study team, facilities in which initial contact with their hospitalised mother took place were described as: “blood-stained room with broken furniture”. Similarly, children who had not visited an inpatient unit were shocked by photos of family rooms, calling the bare spaces “horrible” and “like prison”. Children, carers, and HCPs have identified the negative impact of poor visiting provision on the families of hospitalised parents [9]. Conversely, three reviews of parents’ experiences of hospitalisation both identified improved provision for family visits as a mechanism through which parent-child connection could be improved [10–12].

This study seeks to generate knowledge about family visit provision within inpatient units from the perspective of parents, carers, children and young people and healthcare providers. It will identify environmental and procedural enhancements to promote wellbeing for families during visits. In doing so, it builds upon prior research which has identified deficits in provision for these service users and their families. It will use qualitative interviews to understand the limitations of, and required improvements for provision, drawing upon principles of environmental psychology. In particular, it will employ the Theory of Supportive Design, which seeks to develop environments which reduce stress in users, fostering a perception of control, social support and positive distraction [13]. A subsequent survey will list improvements/components generated in the interviews and ask respondents to rank them in order of priority and consider them in relation to the Theory of Supportive Design. The findings from these two work packages will address a gap in the literature, both in terms of up-to-date assessment of provision, and of targets for enhancement. It is, furthermore, designed to generate data which will contribute to the co-production of a ‘Family Visit Pack’, a set of practice guidelines, and templates to enhance future provision for family visits within the NHS (a second linked project which is not detailed further in this protocol).

## Methods/Materials and methods

This study aims to understand the perceptions and experiences of family hospital visits from the viewpoints of stakeholders and end users (patients, children and young people, carers and healthcare professionals) with the aim of identifying potential enhancements to such visits. This will inform a co-production process to develop a template ‘Family Visit Pack’ (this is a subsequent project which is not detailed in this protocol).

### Design

The study utilises an exploratory sequential mixed method design in which qualitative data collected during online interviews (work package 1) will inform the design of a subsequent questionnaire (work package 2). The results of both will be utilised to inform the co-production process for the Family Visit Pack.

### Study setting

Work Package 1: As the study is recruiting those with current and prior experience of hospitalisation, it will be set in both the NHS and the community. Participant identification and data collection (via video/telephone interviews) will be conducted online, in the community and within participating NHS trusts.

Work Package 2: The questionnaire will be distributed within the community (online) and participating NHS sites (online or paper version).

### Participants and eligibility criteria

#### Sample size.

The sample size for work package 1 (qualitative interviews, N = 36–52: parents = 12–16; carers = 6–10; children and young people = 6–10; healthcare professionals = 12–16) has been derived with consideration of the Information Power Model [14].

The sample size for work package 2 (N = 100: parents = 50; carers = 25; healthcare professionals = 25) reflects the 2007 Barnardo’s audit (parents = 36) and earlier surveys of patient experience and reflects the additional opportunities to recruit afforded by social media [8]. If the upper limit of the target sample is reached, the study team will retain the option to continue to recruit for the duration of the data collection window to enhance data quality and diversity.

#### Eligibility criteria.

The sample will comprise: adults who have experience of inpatient psychiatric hospitalisation and who have dependent children; children and young people who have experienced a parent being hospitalised; carers of children whose parent has been hospitalised; and healthcare professionals with experience of working on an adult IPU (private and NHS provision is eligible). For parents, children and carers of children, the experience of hospitalisation is required to have taken place since July 2021 (the point at which COVID-19 related visiting restrictions ended), whereas for healthcare professionals the period is shorter (2 years) to support clarity of recollection.

For all participant types, within both work packages, a broad and pragmatic set of eligibility criteria has been established, in order to maximise inclusion. No participant will be excluded on the grounds of gender, socio-economic status, clinical presentation, location or ethnicity. Translation and interpreter involvement will be provided, so no exclusion will be made on grounds of language. All participants are required to have access to an electronic device or telephone (with access to internet (and camera for CYPs)) for purposes of interviewing.

See Table 1 for criteria organised according to participant type and work package.

**Table 1:** Inclusion and Exclusion Criteria by Participant Type and Work Package.

Work Package	Participant Type	Inclusion Criteria	Exclusion Criteria
1	Children and Young People	• Aged 8–18 years. • Can recollect visiting a parent (including co-parent/biological/adopted/step/grandparent) who has been treated on a IPU since July 2021. • Capacity to consent to take part (if 16 or over) or assents to take part and parent/carer consents for their participation (ages 8–15). • Has provided contact information for parent/carer.	• Aged under 8 years of age • Difficulties with speech, learning or development such that involvement in the interview process is deemed to be challenging or distressing (will be determined with CYP and carer)
1&2	Parent	• Adults (18 years and above). • Current or prior experience (since July 2021) of psychiatric hospitalisation while a parent (biological, step, adopted) to child (<18 at time of hospitalisation) who visited a parent in hospital. • Capacity to consent.	• Under 18 years of age • Does not have capacity to consent
	Carers	• Adult (18 years and above) • Capacity to consent • Current/prior experience (since July 2021) of caring for a child (<18) whose parent (co-parent/biological parent; grandparent; sibling; foster carer) was hospitalised on IPU.	• Under 18 years of age • Does not have capacity to consent
	Healthcare Professionals	• Adult (18 years and above) • Currently working on an adult IPU or has worked on an adult IPU in the last two years for a minimum of six months. Roles will include nurses, care assistants, psychologists, occupational therapists, psychiatrists, reception staff.	• Works in a role which does not have familiarity with family rooms (e.g., human resources)

#### Capacity.

Capacity to consent will be assessed in line with the Mental Capacity Act (2005) and its Code of Practice, which establish that capacity is decision-specific, can fluctuate, and must be assessed against four key abilities: understanding, retention, weighing up, and communication [15]. This approach reflects the INCLUDE Impaired Capacity to Consent Framework, which promotes structured and ethical inclusion of participants who may have fluctuating or impaired capacity [16]. Capacity will be assessed during the initial introductory conversation with a participant and throughout the interview.

Parents currently receiving inpatient care: Capacity will be jointly assessed by ward staff (e.g., the patient’s named nurse or responsible clinician) and a member of the research team. This dual approach ensures that both clinical knowledge of the participant’s mental state and the researcher’s responsibility for informed consent are taken into account.

#### Recruitment.

To maximise breadth of recruitment, participants will be recruited within the community and within participating trusts within the NHS. Recruitment materials will be distributed through public social media channels (e.g., Instagram), online groups and in collaboration with third sector organisations. Whilst a parent, child and carer could be from the same family, there is no requirement for this form of dyadic recruitment.

#### Ethics approval and consent to participate.

Approval granted 26 January 2026 by East Midlands – Derby Recruitment Ethics Committee [Reference **26/EM/0005].**

All participants aged 16 and over will provide informed consent. Participants aged 8–15 will provide assent and consent will be obtained from their parent/legal guardian.

The planning, conduct and reporting of this study will be in accordance with Good Clinical Practice (GCP), the principles stated in the Declaration of Helsinki (1996) and all applicable regulatory requirements. The involvement of children in the research follows principles outlined in UN Convention on Rights of a Child (UNCRC) and NIHR INVOLVE guidance on the planning, conduct and reporting of this study will be in accordance with Good Clinical Practice (GCP), the principles stated in the Declaration of Helsinki (1996) and all applicable regulatory requirements. The involvement of children in the research follows principles outlined in UN Convention on Rights of a Child (UNCRC) and NIHR INVOLVE guidance on children and young people’s participation.

Should any amendments to the protocol be required, the researcher will contact the University Sponsor to determine if it’s substantial or non-substantial. The amendment will not be submitted to the REC until sponsor approval has been received. Upon clarification of the amendment categorisation, the researcher will submit the amendment as per current UK Health Departments’ Research Ethics Service (the HRA) practice. The amendment will not be implemented until all approvals have been completed.

The researcher will notify the REC of the end of the study within 90 days of the last data collection point using HRA current practice.

### Procedures

#### Work package 1.

All participants will be provided with a participant information sheet (PIS) (including a simplified sheet for CYP participants) which describes the project and what participation involves. All PIS are available for download on the study website (http://inpatientfamilies.org/) [see Additional File 1 for participant information and consent forms] Participants who express interest in taking part (via email, face-to-face, or the website) will be emailed the relevant PIS and have an initial conversation with the research team during which eligibility will be determined. During this conversation, participants will be given the opportunity to ask any questions they might have after reading the PIS.

Parents, carers, HCPs: Will consent using an online informed consent form (ICF) hosted on the Qualtrics survey platform. If preferred, participants can provide consent by email or by using a paper version, which will be posted to them and returned by email or post (to the study team at University of Sussex).

Children and young people: Those aged 15 or younger will be asked to provide assent and their parent/carer will provide consent. In both cases, this will be via an online form, email, or paper version, following the procedure above. CYPs aged 16 and above will be invited to consent.

Interviews (30–60mins), will either take place online (all participants), via telephone (adult participants only) or in person (adult participants currently admitted to an inpatient unit or staff working on an inpatient unit at one of the NHS research sites). Adult interviews will be conducted by up to two members of the team, ideally including one lived-experience researcher. CYPs along with their carer will decide whether the CYP should be supported by parent/carer during the interview. Interviews will be recorded (audio and/or audio and video) within Microsoft Teams, after which transcripts will be downloaded and stored on a secure University of Sussex server. At this point the origin data files will be deleted, and the transcripts will then be anonymised.

At the start of the interview, participants will be reminded of their right to anonymity, take break or stop and verbal consent will be requested. Interviews will be recorded (audio and/or audio and video) within Microsoft Teams, after which transcripts will be downloaded and stored on a secure University of Sussex server.

**Fig 1:**
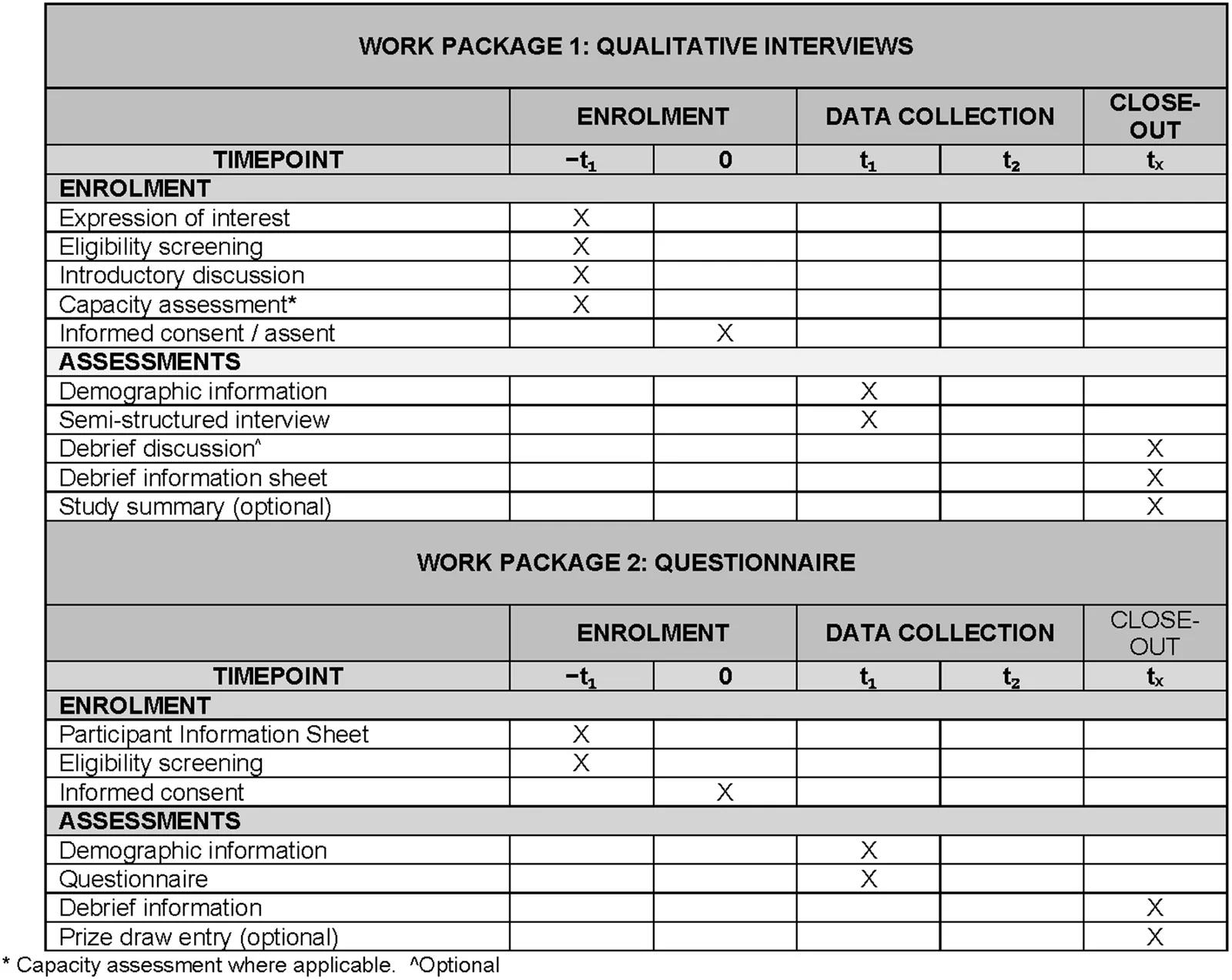
SPIRIT 2025 Checklist: Schedule of Enrolment and Assessment.

At the end of the interview, the recording will be stopped, and the participant will be asked if they would like to have a short debrief session with the lived experience researcher who co-conducted the interview. For CYP participants, this will be with the research team interviewer. This will take the form of a short informal grounding conversation in which the participant has space to come back into the present moment. This is to give the participant time to reflect and ground themselves and is optional. Additionally, participants can arrange an optional follow-up call to take place with a researcher in the days following the interview. After the interview, participants will be provided with the debrief sheet and parent, CYP, and carer participants will be issued a £10 voucher. See Fig 1. for Spirit 2025 schedule [17].

#### Work package 2.

Participation can take place online; participating NHS sites can also choose to administer paper versions of the questionnaires. Online participation: following provision of the PIS and online eligibility screening, participants will provide consent using an online Qualtrics form after which they will gain access to the questionnaire. Participants will be able to download a copy of the PIS. After the completion of the online questionnaire, participants will be able to download the debrief sheet and can submit their email address through a separate Qualtrics questionnaire to win a £50 voucher.

Participation through paper version within NHS trusts: eligible participants will be provided with a physical copy of the PIS and provide consent with a paper version of the ICF, then they will be provided with the paper version of the questionnaire. Participants will then be provided a copy of the debrief sheet and asked whether they want to submit their email to win a £50 voucher.

Staff will collect, scan and then securely destroy the paper ICF and questionnaires, scanned copies will be sent to the research team through the encrypted NHS email service. Staff will submit the email addresses of participants wanting to take part in the prize draw to the relevant Qualtrics questionnaire. Research staff will enter questionnaire answers into the Qualtrics data, scans of the ICFs will be stored on a secure University of Sussex server.

### Measures

#### Work package 1.

Parent, carer and HCP will partake in semi-structured interviews [see Additional File 1 for topic guides]. The topic guides for these interviews are designed to gather information focused on the following core areas: (1) Demographic questions about the participant and their family. (2) How did/do participants experience family-visit provision? (3) How would they improve aspects of provision (e.g., family room information provided)? (4) During family visits, what changes/actions/elements would support parents/children to: (a) Feel in control of the situation? (b) Provide positive distraction? (c) Increase family connection and opportunities for social support? (5) How could wellbeing be supported during family visits?

The interviews with children and young people will use a “draw, write, tell” approach which has been identified as facilitating involvement in interviews from children with a broad range of ages and abilities, as well as those with limited English-speaking skills [18]. The online interview approach will take the following format, which reflects the core elements of the approach developed by Angell and Angell [18]. From the outset, the interviewer will seek to build rapport with the CYP to support an effective working relationship and maximise the time together. After brief familiarisation with the drawing tools, the CYP is invited to remember their experience of visiting their parent in hospital and to draw whatever those memories made them think about. They are told they can add writing to their drawing if they wish to. They are advised that there are no right or wrong answers. Once the drawing is complete, the participant is invited to describe their drawing using open-ended questions. Questions relating to how hospital visitation could be improved for CYPs will be asked if this is not part of the above discussion. If the CYP consents, their ‘drawings’ are downloaded or captured through a screenshot.

#### Work package 2.

The brief anonymous questionnaires focus on the experience of family visits and suggested enhancements [see Additional file 1]. The questionnaire will be developed by the study team and will integrate data from Work Package 1. It will include a list of improvements/components generated in response to the findings of Phase 1. These will be developed from the point of data familiarisation onwards (see Analysis section for further information).

The questionnaire will use these items to engage with Ullrich’s [13] core elements of supportive design (control, positive distraction and social support). This will take the form of inviting participants to match specific emotions to potential enhancements: e.g., “If there were [clearer signage]/ [artwork]/ [comfortable furniture] I would feel… in control/ less anxious/ more relaxed/ more connected with my family member…”. Participants will also be asked to rank proposed improvements in order of priority. Additional items will incorporate salient concepts from environmental psychology and supportive healthcare design – e.g., reducing exposure to environmental stress [19].

This will take the form of inviting participants to match specific emotions (happy, relaxed, calm, safe, interested, energised, in control, connected to others, other) to potential enhancements (e.g., information about how to arrange/book visit).

They will also be asked to rank improvements in order of priority. This approach will facilitate validation of work package 1 findings and use a larger dataset to prioritise recommendations for enhancements generated during qualitative data collection [20].There will also be optional free text boxes for participants to provide recommended enhancement to provision – an approach which has been found to improve the quality and quantity of data related to satisfaction with services in questionnaire-based methods [21]. Face validity of the questionnaire will be assessed by the Lived Experience Advisory Panel attached to the study, and relevant stakeholders (e.g., healthcare professionals).

All items, excluding the optional free-text question, will be forced response to maximise data collection. The demographic, family background, service use/professional practice will include a ‘prefer not to answer’ option.

### Analysis

#### Work package 1.

The analysis will be conducted to meet two objectives: 1) to describe participants’ experiences of hospital visitation; 2) to generate a list of potential components/enhancements for provision to incorporate into the questionnaire deployed in work package 2, which will be used in subsequent co-design work.

Interviews will be transcribed by the research team, and subject to a five-stage framework analysis (in NVivo) which involves familiarisation, theme identification, indexing (the application of themes onto transcripts), charting and summarising using a matrix, and finally interpretation/mapping. This analytical approach is well-suited to the project’s research objectives, as it is flexible; can accommodate large and relatively non-homogenous datasets, including those from varied participant types; and enables the inclusion of themes developed from the data alongside a priori concepts (e.g., suggested environmental improvements to enhance control, positive distraction and connection). The list of components for work package 2 will be generated during the familiarisation and theme identification stages, which will facilitate timely progression into work package 2 data collection. Transcription and analysis will occur concurrently with data collection to expedite generating the results. Analysis will be carried out by the research team and lived experience researchers will inform and review the analysis throughout.

#### Work package 2.

All analysis will be conducted using SPSS and/or R(Studio). Quantitative analysis will be descriptive in nature, as the study is exploratory and not powered for inferential statistics. Descriptive statistics will be used to summarise the demographics and questionnaire responses, specifically: (1) Frequencies and percentages will be reported for categorical variables (e.g., endorsement of each emotion linked to environmental enhancements). (2) Means, medians, and standard deviations will be used to summarise continuous or ordinal variables (e.g., average ranking positions of enhancements). (3) For the ranking exercise, mean rank scores will be calculated alongside frequency counts of “top five” selections, to provide indications of both central tendency and practical salience. (4) Subgroup trends (parents, carers, HCPs) will be described but not subjected to hypothesis testing.

Free-text qualitative data will be incorporated into the above framework analysis (work package 1) in NVivo and used to generate a word cloud. Analysis will be conducted by the research team, including lived experience researchers.

#### Status and timeline of the study.

Recruitment for the project began on 03^rd^ March 2026 with data collection continuing until 30^th^ November 2026. Recruitment and data collection for WP1 will run from March 2026 to August 2026 and recruitment and data collection for WP2 will run from June 2026–30^th^ November 2026. It is expected that results will be completed by February 2027. See Table 2. for SPIRIT Timeline

**Table 2:** SPIRIT participant timeline.

WORK PACKAGE 1: QUALITATIVE INTERVIEWS					
	**ENROLMENT**		**DATA COLLECTION**		**CLOSE-OUT**
**TIMEPOINT**	**−t_1_**	**0**	**t_1_**	**t_2_**	**tₓ**
**ENROLMENT**					
Expression of interest	X				
Eligibility screening	X				
Introductory discussion	X				
Capacity assessment*	X				
Informed consent/ assent		X			
**ASSESSMENTS**					
Demographic information			X		
Semi-structured interview			X		
Debrief discussion^^^					X
Debrief information sheet					X
Study summary (optional)					X
**WORK PACKAGE 2: QUESTIONNAIRE**					
	**ENROLMENT**		**DATA COLLECTION**		CLOSE-OUT
**TIMEPOINT**	**−t_1_**	**0**	**t_1_**	**t_2_**	**tₓ**
**ENROLMENT**					
Participant Information Sheet	X				
Eligibility screening	X				
Informed consent		X			
**ASSESSMENTS**					
Demographic information			X		
Questionnaire			X		
Debrief information					X
Prize draw entry (optional)					X

* Capacity assessment where applicable. ^Optional.

## Discussion

This study aims to address a significant gap in understanding the experiences of families when a parent is admitted to psychiatric inpatient care. By incorporating perspectives from parents, children and young people, carers, and healthcare professionals, the study seeks to generate a comprehensive understanding of current provision and priorities for improvement, which will inform the co-production of a ‘Family Visit Pack’ of guidance principles and environmental enhancements designed to improve visits.

This study will recruit participants from the community (e.g., social media, communities of interest, and charities) and five collaborating NHS sites. Consequently, the findings may not fully capture experiences across other NHS sites, private mental health settings, or across demographic groups.

On completion of the study, the data will be analysed and tabulated and a Final Study Report prepared for REC and Sponsor. A final accessible lay study report will be produced and published on the study website and shared with participants who wish to receive it. Reporting of study findings will use the appropriate guidelines as identified from the EQUATOR network (https://www.equator-network.org/).

### Amendments

**Table:** 

*Protocol version no.*	*Date issued*	*Author(s) of changes*	*Details of changes made*
*2.1*	*13 Feb 26*	*CI*	Inclusion of demographic questions on CYP topic guide; inclusion of geographical identifier (county) on adult topic guides and questionnaires; general (all groups) recruitment poster./
*2.1*	*24 March 26*	*CI*	An additional data field requesting information about which county the participant resides in has been added to the online questionnaire used for all WP2 participants
*2.1*	*25 March 26*	*CI*	Addition of new site: Somerset Foundation NHS Trust.
*2.4 (2.2 and 2.3 related to non-agreed changes which were incorporated into v 2.4*	*28 April 26*	*CI*	Addition of new sites: North staffs and Lincolnshire
*2.5*	*10 June 26*	*CI*	Paper questionnaires and new questionnaire version

## Supporting information

S1 File POPI Materials. This contains all participant materials, interview schedules and questionnaires.
(PDF)

S2 File POPI_SPIRIT. The SPIRIT checklist for the POPI protocol.
(PDF)
