# Supplementary material for: Mixed methods investigation into provision of family rooms and support for parents admitted to psychiatric inpatient units and their children: Study protocol
Source: PLoS One. 2026 Sep 15;21(9):e0355693. doi: 10.1371/journal.pone.0355693 (PMC13577376; doi:10.1371/journal.pone.0355693)
Supplement: Supplementary file 1 [file pone.0355693.s001.pdf]

POPI Materials

This document includes supplementary materials for the Provision for Parents on Psychiatric Inpatient Units (POPI) study,

Participant materials: Participant Information Sheets, Information and Consent Forms, Assent Forms.

Administration materials: Interview Topic Guides and Questionnaires used in the POPI study.

Abbreviations:

HCP – Healthcare Professionals

CYP – Children and Young People

PIS - Participant Information Sheet

ICF - Information and Consent Forms

WP1 – Work package 1

WP2 – Work Package 2

Contents

WP1 Parent PIS	3
WP1 Carer PIS	8
WP1 HCP PIS	13
WP1 CYP Carer PIS	18
WP1 CYP 8-11 PIS	23
WP1 CYP 12-15 PIS	25
WP1 CYP 16-18 PIS	30
WP1 Parent ICF	35
WP1 Carer ICF	37
WP1 HCP ICF	39
WP1 CYP Carer ICF	41
WP1 CYP 8-11 Assent	43
WP1 CYP 12-15 Assent	44
WP1 CYP 16-18 ICF	45
WP1 Parent Topic Guide	47
WP1 Carer Topic Guide	50
WP1 HCP Topic Guide	53
WP1 CYP Topic Guide	55
WP2 Online PIS	58
WP2 Paper PIS	63
WP2 Online ICF	68
WP2 Parent Questionnaire	69
WP2 Carer Questionnaire	75
WP2 HCP Questionnaire	84

PARTICIPANT INFORMATION SHEET (PARENT) NHS

Title of Study: Mixed methods investigation into provision of family rooms and support for parents admitted to psychiatric inpatient units and their children.

PLEASE KEEP A COPY OF THIS INFORMATION SHEET FOR YOUR RECORDS

Section: Taking Part

Invitation Paragraph

We would like to invite you to participate in this research project. You should only participate if you want to; choosing not to take part will not disadvantage you in any way. Before you decide whether you want to take part, it is important for you to understand why the research is being done and what your participation will involve. Please discuss the study with others if you wish. If you have any questions you can contact us using the contact details at the end of this information sheet.

What is the purpose of the study?

The aim of this study is to understand the experience of family visits when a parent is treated in a mental health inpatient hospital.
We are specifically interested in how we can improve the experience of these visits for parents and their children.
This will involve carrying out interviews with parents, children and young people, carers of children who visited a parent in hospital and healthcare professionals.

Who is responsible for this study?

[Placeholder]

Why have I been invited to take part?

You are invited to participate in this study because you are a parent (including foster, step-parent, adopted or grandparent with primary care responsibility) of a child 18 or under. You are also currently staying on a mental health inpatient unit or have stayed on one since July 2021.

Do I have to take part?

Participation is voluntary and you do not have to take part. We will describe the study in this information sheet and will give you 7 days to read this, so you can decide whether you

wish to take part in this study. Please contact us if there is anything that is not clear, if you have any questions, or if you would like more information.

<u>What will happen to me if I decide to take part?</u>

If you decide to take part, you will be given this information sheet to keep and will be invited to have a short conversation with the research team where you can find out more about the project and we can decide whether it is a good fit for you. If you are currently receiving treatment in hospital, you can ask for a member of the care team to contact the research team on your behalf and help you arrange a call or meeting with them. After that, you will be asked to sign an online consent form to confirm your agreement to participate. You will be given a copy of this consent form to keep.

You will then take part in an interview. This interview will take place online or over the telephone. If you are currently within an inpatient unit ([Placeholder]) this interview can also take place face-to-face at the hospital. The interviews will be scheduled at a convenient time for you. The interview will be audio/video-recorded. An anonymised (with all identifying data removed) transcript of your interview will be produced (automatic transcription through Microsoft teams, transcription software, and/or transcription by a researcher), after which the audio recording of your interview will be deleted.

After the interview you will be invited to have an informal debrief conversation.

<u>What happens if I do not want to take part or if I change my mind?</u>

You are free to withdraw from the study at any time, without giving a reason. You can withdraw your data (information about you and the transcript of the recording of the interview) up to the point at which it is anonymised (one month after the interview takes place).

<u>What are the possible benefits in taking part?</u>

Your insights will contribute to improving the experiences of patients who are parents and families visiting psychiatric units. As a thank you for your time we will provide a £10 voucher after the interviews have taken place.

<u>Are there any potential risks involved?</u>

A possible disadvantage to taking part in the study is that some interview questions may prompt reflection on difficult experiences. In order to reduce any potential risks, you are not required to answer any question you find uncomfortable. A debrief and signposting to support resources will be provided.

Are there limits to confidentiality?

Should you disclose information which indicates you or someone else is at risk of harm we may have to contact the appropriate services. In this case we will have to disclose your identity.

How is the project being funded?

[Placeholder]

What will happen to the results of the study?

We will produce a final report summarising the main findings. This will be available on our project website [Placeholder].

This research may be published in peer reviewed academic journals under open access conditions. This means the article can be read for free online by anyone who wishes to. The findings will be presented to stakeholder groups and at conferences.

Anonymised quotes from your interview may be used in these publications.

You will be sent a summary of the results of the study if you indicate you would like to receive one.

Who has reviewed this study?

This research has been reviewed by an independent group of people, called an Ethics Committee. This study was reviewed and given a favourable opinion by the [Placeholder] which indicates it is ethical, safe and respects the rights of people taking part.

Section: Your personal data

How will we use information about you?

We will need to use information from you for this research project.

This information will include:
- your name
- your contact details.

People will use this information to do the research or to check your records to make sure that the research is being done properly.

People who do not need to know who you are will not be able to see your name or contact details. Your data will have a code number instead.

[Placeholder] is responsible for looking after your information. We will share your information related to this research project with the following types of organisations:

- Regulatory bodies and ethics committees
- Other researchers and institutions (in an anonymised form where you cannot be identified).

We will keep all information about you safe and secure by:

- Anonymising the data
- Keeping any identifiable information in a separate password protected file
- Storing information about you on a secure [Placeholder] -hosted online platform.

Your data will not be shared outside the UK.

How will we use information about you after the study ends?

Once we have finished the study, we will keep some of the data so we can check the results. We will write our reports in a way that no-one can work out that you took part in the study.
We will keep your study data for a maximum of 10 of years. The study data will then be fully anonymised and securely archived or destroyed. Identifiable data will be retained for a maximum of 12 months post-study on a secure university server before being destroyed.

What are your choices about how your information is used?

You can stop being part of the study at any time, without giving a reason, but we will keep information about you that we already have.

You have the right to ask us to access, remove, change or delete data we hold about you for the purposes of the study. You can also object to our processing of your data. We might not always be able to do this if it means we cannot use your data to do the research. If so, we will tell you why we cannot do this.

If you agree to take part in this study, you will have the option to take part in future research using your data saved from this study. To facilitate this, your anonymised data will be stored in the Figshare data repository where it can be accessed by other researchers in an anonymised form.

Where can you find out more about how your information is used?

You can find out more about how we use your information as follows:

[Placeholder]

Section: Further information

What if you have a query or something goes wrong?

If you are unsure about something you can contact the research team for further advice using the contact details at the bottom of this information sheet.

However, if your query has not been handled to your satisfaction, or if you are unhappy and wish to make a formal complaint to someone independent of the research team, then please contact:

[Placeholder]

You can also follow the NHS Complaints Procedure.

[Placeholder]

If you wish to complain or have any concerns about any aspect of the way you have been treated during the course of this study then you should follow the instructions given above.

Support if you're feeling upset or distressed

We know that thinking about difficult experiences can sometimes create upsetting feelings. To help you look after yourself, we suggest you please consider making use of any things you usually find helpful. Suggestions from previous participants include:

- Contacting a friend, family member or support worker afterwards
- Having your pet nearby
- Using quiet fidget toys or stimming aids
- Spending time in a comforting space or doing an activity that soothes you (e.g., music, a walk, favourite TV show)

If you feel you would benefit from additional support, the following organisations may be helpful:
Support for parents and carers:

- Mind: 0300 123 3393 or www.mind.org.uk
- Rethink Mental Illness: 0808 801 0525 or www.rethink.org
- Family Lives (parenting support): 0808 800 2222 or www.familylives.org.uk
- YoungMinds Parent Helpline: 0808 802 5544 or www.youngminds.org.uk

Who should I contact for further information?

If you have any questions or require more information about this study, please contact me using the following contact details:

[Placeholder]

Thank you for reading this information sheet and for considering taking part in this research.

PARTICIPANT INFORMATION SHEET (CARER) NHS

Title of Study: Mixed methods investigation into provision of family rooms and support for parents admitted to psychiatric inpatient units and their children.

PLEASE KEEP A COPY OF THIS INFORMATION SHEET FOR YOUR RECORDS

Section: Taking Part

Invitation Paragraph

We would like to invite you to participate in this research project. You should only participate if you want to; choosing not to take part will not disadvantage you in any way. Before you decide whether you want to take part, it is important for you to understand why the research is being done and what your participation will involve. Please discuss the study with others if you wish. If you have any questions, you can contact us using the contact details at the end of this information sheet.

What is the purpose of the study?

The aim of this study is to understand the experience of family visits when a parent is treated in a mental health inpatient hospital.
We are specifically interested in how we can improve the experience of these visits for parents and their children,
This will involve carrying out interviews with parents, children and young people, carers of children who visited a parent in hospital and healthcare professionals.

Who is responsible for this study?

[Placeholder]

Why have I been invited to take part?

You have been invited to participate in this study because you currently care for or have recently cared for (since July 2021), a child whose parent has been admitted to a psychiatric hospital.

Do I have to take part?

Participation is voluntary and you do not have to take part. We will describe the study in this information sheet and will give you 7 days to read this, so you can decide whether you wish to take part in this study. Please contact us if there is anything that is not clear, if you have any questions, or if you would like more information.

What will happen to me if I decide to take part?

If you decide to take part, you will be given this information sheet to keep and will be invited to have a short conversation with the research team where you can find out more about the project and we can decide whether it is a good fit for you. After that, you will be asked to sign an online consent form to confirm your agreement to participate. You will be given a copy of this consent form to keep.

You will then take part in an interview (online or over the telephone). The interviews will be scheduled at a convenient time for you. The interview will be recorded (audio/video).
An anonymised (with all identifying data removed) transcript of your interview will be produced (automatic transcription through Microsoft teams, transcription software, and/or transcription by a researcher), after which the audio recording of your interview will be deleted.

After the interview you take part in an optional informal debrief session.

What happens if I do not want to take part or if I change my mind?

You are free to withdraw from the study at any time, without giving a reason. You can withdraw your data (information about you and the transcript of the recording of the interview) up to the point at which it is anonymised (one month after the interview takes place)

What are the possible benefits in taking part?

Your insights will contribute to improving the experiences of patients who are parents and families visiting psychiatric units. As a thank you for your time we will provide a £10 voucher after the interviews have taken place.

Are there any potential risks involved?

A possible disadvantage to taking part in the study is that some interview questions may prompt reflection on difficult experiences. In order to reduce any potential risks, you are not required to answer any question you find uncomfortable. A debrief sheet providing information about what happens next in the project along with signposting to support resources will be provided.

Are there limits to confidentiality?

Should you disclose information which indicates you or someone else is at risk of harm we may have to contact the appropriate services. In this case we will have to disclose your identity.

How is the project being funded?

[Placeholder]

What will happen to the results of the study?

We will produce a final report summarising the main findings. This will be available on our project website [Placeholder].

This research may be published in peer reviewed academic journals under open access conditions. This means the article can be read for free online by anyone who wishes to. The findings will be presented to stakeholder groups and at conferences.

Anonymised quotes from your interview may be used in these publications.

You will be sent a summary of the results of the study if you indicate you would like to receive one.

Who has reviewed this study?

This research has been reviewed by an independent group of people, called an Ethics Committee. This study was reviewed and given a favourable opinion by the [Placeholder] which indicates it is ethical, safe and respects the rights of people taking part.

Section: Your personal data

How will we use information about you?

We will need to use information from you for this research project.

This information will include:

- your name
- your contact details.

People will use this information to do the research or to check your records to make sure that the research is being done properly.

People who do not need to know who you are will not be able to see your name or contact details. Your data will have a code number instead.

[Placeholder]is responsible for looking after your information. We will share your information related to this research project with the following types of organisations:

- Regulatory bodies and ethics committees
- Other researchers and institutions (in an anonymised form where you cannot be identified).

We will keep all information about you safe and secure by:

- Anonymising the data
- Keeping any identifiable information in a separate password protected file
- Storing information about you on a secure [Placeholder]-hosted online platform.

Your data will not be shared outside the UK.

How will we use information about you after the study ends?

Once we have finished the study, we will keep some of the data so we can check the results. We will write our reports in a way that no-one can work out that you took part in the study.
We will keep your study data for a maximum of 10 of years. The study data will then be fully anonymised and securely archived or destroyed. Identifiable data will be retained for a maximum of 12 months post-study on a secure university server before being destroyed.

What are your choices about how your information is used?

You can stop being part of the study at any time, without giving a reason, but we will keep information about you that we already have.

You have the right to ask us to access, remove, change or delete data we hold about you for the purposes of the study. You can also object to our processing of your data. We might not always be able to do this if it means we cannot use your data to do the research. If so, we will tell you why we cannot do this.

If you agree to take part in this study, you will have the option to take part in future research using your data saved from this study. To facilitate this, your anonymised data will be stored in the Figshare data repository where it can be accessed by other researchers in an anonymised form.

Where can you find out more about how your information is used?

You can find out more about how we use your information as follows:

- [Placeholder]

Section: Further information

What if you have a query or something goes wrong?

If you are unsure about something you can contact the research team for further advice using the contact details at the bottom of this information sheet.

However, if your query has not been handled to your satisfaction, or if you are unhappy and wish to make a formal complaint to someone independent of the research team, then please contact:

[Placeholder]

If you wish to complain or have any concerns about any aspect of the way you have been treated during the course of this study then you should follow the instructions given above.

You can also follow the NHS Complaints Procedure.
[Placeholder]

Who should I contact for further information?

If you have any questions or require more information about this study, please contact me using the following contact details:

[Placeholder]

Support if you're feeling upset or distressed

We know that thinking about difficult experiences can sometimes create upsetting feelings. To help you look after yourself, we suggest you please consider making use of any things you usually find helpful. Suggestions from previous participants include:

- Contacting a friend, family member or support worker afterwards
- Having your pet nearby
- Using quiet fidget toys or stimming aids
- Spending time in a comforting space or doing an activity that soothes you (e.g., music, a walk, favourite TV show)

If you feel you would benefit from additional support, the following organisations may be helpful:
Support for parents and carers:

- Mind: 0300 123 3393 or www.mind.org.uk
- Rethink Mental Illness: 0808 801 0525 or www.rethink.org
- Family Lives (parenting support): 0808 800 2222 or www.familylives.org.uk
- YoungMinds Parent Helpline: 0808 802 5544 or www.youngminds.org.uk

Thank you for reading this information sheet and for considering taking part in this research.

PARTICIPANT INFORMATION SHEET (HCP) NHS

Title of Study: Mixed methods investigation into provision of family rooms and support for parents admitted to psychiatric inpatient units and their children.

PLEASE KEEP A COPY OF THIS INFORMATION SHEET FOR YOUR RECORDS

Section: Taking Part

Invitation Paragraph

We would like to invite you to participate in this research project. You should only participate if you want to; choosing not to take part will not disadvantage you in any way. Before you decide whether you want to take part, it is important for you to understand why the research is being done and what your participation will involve. Please discuss the study with others if you wish. If you have any questions you can contact us using the contact details at the end of this information sheet.

What is the purpose of the study?

This study aims to explore how families experience visiting a parent in a psychiatric hospital. We are particularly interested in the views of healthcare professionals who work on adult inpatient units, to understand current practice and how the environment or procedures could be improved. Findings will inform the development of a 'Family Visit Pack' – a set of guidelines, templates, and resources to support visiting arrangements and promote wellbeing during family visits.

Who is responsible for this study?

[Placeholder]

Why have I been invited to take part?

You have been invited to participate in this study because you currently work, or have recently worked (within the past two years for a minimum of six months), on an adult psychiatric inpatient unit and are familiar with family visiting practices.

Do I have to take part?

Participation is voluntary. You will have at least 7 days to review this information sheet before deciding. You are free to ask any questions or request additional information. Please contact us if there is anything that is not clear, if you have any questions, or if you would like more information.

What will happen to me if I decide to take part?

If you decide to take part, you will be given this information sheet to keep and will be asked to sign an online consent form to confirm your agreement to participate. You will be given a copy of this consent form to keep.

We will then invite you to take part interview (online or over the telephone). The interviews will be scheduled at a convenient time for you. The interviews will be scheduled at a convenient time for you. The interview will be audio/video-recorded. An anonymised (with all identifying data removed) transcript of your interview will be produced (automatic transcription through Microsoft teams, transcription software, and/or transcription by a researcher), after which the audio recording of your interview will be deleted.

After the interview there is an optional debrief conversation.

What happens if I do not want to take part or if I change my mind?

You are free to withdraw from the study at any time, without giving a reason. You can withdraw your data (information about you and the transcript of the recording of the interview) up to the point at which it is anonymised (one mont after the interview takes place)

What are the possible benefits in taking part?

While there are no direct personal benefits, your insights will contribute to improving the experiences of patients who are parents and families visiting psychiatric units.

Are there any potential risks involved?

A possible disadvantage to taking part in the study is that some interview questions may prompt reflection on difficult experiences. In order to reduce any potential risks, you are not required to answer any question you find uncomfortable. Debriefing information and signposting to support resources will be provided.

Are there limits to confidentiality?

Should you disclose information which indicates you or someone else is at risk of harm we may have to contact the appropriate services. In this case we will have to disclose your identity.

How is the project being funded?

[Placeholder]

What will happen to the results of the study?

We will produce a final report summarising the main findings. This will be available on our project website [Placeholder].
This research may be published in peer reviewed academic journals under open access conditions. This means the article can be read for free online by anyone who wishes to. The findings will be presented to stakeholder groups and at conferences.

Anonymised quotes from your interview may be used in these publications.

You will be sent a summary of the results of the study if you indicate you would like to receive one.

Who has reviewed this study?

This research has been reviewed by an independent group of people, called an Ethics Committee. This study was reviewed and given a favourable opinion by the [Placeholder] which indicates it is ethical, safe and respects the rights of people taking part.

Section: Your personal data

What is personal data?

How will we use information about you?

We will need to use information from you for this research project.

This information will include:
- your name
- your contact details.

People will use this information to do the research or to check your records to make sure that the research is being done properly.

People who do not need to know who you are will not be able to see your name or contact details. Your data will have a code number instead.

[Placeholder] is responsible for looking after your information. We will share your information related to this research project with the following types of organisations:
- Regulatory bodies and ethics committees
- Other researchers and institutions (in an anonymised form where you cannot be identified).

We will keep all information about you safe and secure by:
- Anonymising the data
- Keeping any identifiable information in a separate password protected file
- Storing information about you on a secure [Placeholder] -hosted online platform.

Your data will not be shared outside the UK.

How will we use information about you after the study ends?

Once we have finished the study, we will keep some of the data so we can check the results. We will write our reports in a way that no-one can work out that you took part in the study.
We will keep your study data for a maximum of 10 of years. The study data will then be fully anonymised and securely archived or destroyed. Identifiable data will be retained for a maximum of 12 months post-study on a secure university server before being destroyed.

What are your choices about how your information is used?

You can stop being part of the study at any time, without giving a reason, but we will keep information about you that we already have.

You have the right to ask us to access, remove, change or delete data we hold about you for the purposes of the study. You can also object to our processing of your data. We might not always be able to do this if it means we cannot use your data to do the research. If so, we will tell you why we cannot do this.

If you agree to take part in this study, you will have the option to take part in future research using your data saved from this study. To facilitate this, your anonymised data will be stored in the Figshare data repository where it can be accessed by other researchers in an anonymised form.

Where can you find out more about how your information is used?

You can find out more about how we use your information as follows:

[Placeholder]

Section: Further information

What if you have a query or something goes wrong?

If you are unsure about something you can contact the research team for further advice using the contact details at the bottom of this information sheet.

However, if your query has not been handled to your satisfaction, or if you are unhappy and wish to make a formal complaint to someone independent of the research team, then please contact:

[Placeholder]

If you wish to complain or have any concerns about any aspect of the way you have been treated during the course of this study then you should follow the instructions given above.

You can also follow the NHS Complaints Procedure.

[Placeholder]

Who should I contact for further information?

If you have any questions or require more information about this study, please contact me using the following contact details:

[Placeholder]

Support and resources

If you feel you would benefit from support, the following organisations may be helpful:

Support for healthcare professionals:
[Placeholder]

Thank you for reading this information sheet and for considering taking part in this research.

PARTICIPANT INFORMATION SHEET (CARER CYP) NHS

Title of Study: Mixed methods investigation into provision of family rooms and support for parents admitted to psychiatric inpatient units and their children.

PLEASE KEEP A COPY OF THIS INFORMATION SHEET FOR YOUR RECORDS

Section: Taking Part

Invitation Paragraph

We are inviting the child/young person you care for take part in this research project. We are sharing information about the project with you so you can discuss it with them and help them decide if they want to take part. If they are aged 15 or under, we will ask you to consent for them to take part, so it is important that you understand the project too. Please discuss the study with others if you wish. If you have any questions you can contact us using the contact details at the end of this information sheet.

What is the purpose of the study?

The aim of this study is to understand the experience of family visits when a parent is treated in a mental health inpatient hospital.
We are specifically interested in how we can improve the experience of these visits for parents and carers and children.
This will involve carrying out interviews with children and young people, parents, carers of children who visited a parent in hospital and healthcare professionals.

Who is responsible for this study?

[Placeholder]

Why has the child I care for been invited to take part?

They have been invited to participate because they visited their parent during their stay in an inpatient mental health hospital.

Do they have to take part?

Participation is voluntary and they do not have to take part. We will describe the study in this information sheet and then you will have a week to think about its contents with the child you care for. Please contact us if there is anything that is not clear, if you have any questions, or if you would like more information.

What does taking part involve?

If you decide to take part, you will be given this information sheet to keep and you and the child you care for will be invited to have a short conversation with the research team where you can find out more about the project and we can decide whether it is a good fit for you. After that, you will be asked to sign an online consent form to confirm your agreement for them to participate. They will be asked to sign a form assenting to take part. You will be given a copy of these forms to keep. The child you care for will then take part in an online interview. The interviews will be scheduled at a convenient time for you.

The interview will involve asking the child you care for to draw a picture which reflects their visit to hospital – it can take any form they wish. They can do this using an online drawing tool or on paper. We will ask them to show us the picture and describe it. We will also ask them questions about visiting their parent in hospital and how it could be improved.

The interview will be video-recorded and later transcribed by the research team for analysis. After the interview an anonymised (with all identifying data removed) transcript of the interview will be produced (automatic transcription through Microsoft teams, transcription software, and/or transcription by a researcher), after which the audio recording of the interview will be deleted.

What happens if the child I care for does not want to take part or if they change their mind?

They are free to withdraw from the study at any time, without giving a reason. You/they can withdraw their data (information about you/the child and the transcript of the recording of the interview) up to the point at which it is anonymised (one month after the interview takes place).

What are the possible benefits in taking part?

We hope that it will be a positive experience for the child you care for. Their input will contribute to improving the experiences of families visiting mental health inpatient units. To thank them for their time we give them a £10 voucher after the interviews have taken place.

Are there any potential risks involved?

A possible disadvantage to taking part in the study is that thinking about visiting a parent in hospital might be upsetting. To reduce the risk of this happening we are asking quite broad questions and we will remind all children and young people taking part that they don't have to answer any questions which make them feel uncomfortable. We have written the questions with children who have had similar experiences. We will also encourage the child you care for to bring anything to the interview which makes them feel more comfortable. A debrief sheet providing information about what happens next in the project along with signposting to support resources will be provided.

Are there limits to confidentiality?

Should the child you care for disclose information which indicates they themself or someone else is at risk of harm we may have to contact the appropriate services. In this case we will have to disclose their identity. We would discuss this with you first.

How is the project being funded?

[Placeholder]

What will happen to the results of the study?

We will produce a final report summarising the main findings. This will be available on our project website [Placeholder].

This research may be published in peer reviewed academic journals under open access conditions. This means the article can be read for free online by anyone who wishes to. The findings will be presented to stakeholder groups and at conferences.

Anonymised quotes from the interview may be used in these publications.

You will be sent a summary of the results of the study if you indicate you would like to receive one.

Who has reviewed this study?

This research has been reviewed by an independent group of people, called an Ethics Committee. This study was reviewed and given a favourable opinion by the [Placeholder] which indicates it is ethical, safe and respects the rights of people taking part.

Section: Your personal data

How will we use information about you or the child you care for?

We will need to use information from you/the child for this research project.

This information will include:
- The name of the child you care for
- your contact details.

People will use this information to do the research or to check your records to make sure that the research is being done properly.

People who do not need to know who you are will not be able to see the name of the child you care for or your contact details. The data will have a code number instead.

[Placeholder] is responsible for looking after this information. We will share your information related to this research project with the following types of organisations:

- Regulatory bodies and ethics committees
- Other researchers and institutions (in an anonymised form where you cannot be identified).

We will keep all information about you/the child you care for safe and secure by:

- Anonymising the data
- Keeping any identifiable information in a separate password protected file
- Storing information about you on a secure [Placeholder]-hosted online platform.

Your/the child's data will not be shared outside the UK.

How will we use information about you or the child you care for after the study ends?

Once we have finished the study, we will keep some of the data so we can check the results. We will write our reports in a way that no-one can work out that you or the child that you care for took part in the study.
We will keep study data for a maximum of 10 years. The study data will then be fully anonymised and securely archived or destroyed. Identifiable data will be retained for a maximum of 12 months post-study on a secure university server before being destroyed.

What are your choices about how information about you or the child you care for is used?

The child that you care for can stop being part of the study at any time, without giving a reason, but we will keep information about them that we already have.

You have the right to ask us to access, remove, change or delete data we hold about you for the purposes of the study. You can also object to our processing of your data. We might not always be able to do this if it means we cannot use your data to do the research. If so, we will tell you why we cannot do this.

If you agree to take part in this study, you will have the option to take part in future research using your data saved from this study. To facilitate this, your anonymised data will be stored in the Figshare data repository where it can be accessed by other researchers in an anonymised form.

Where can you find out how the information about you or the child you care for is used?

You can find out more about how we use the information about you or the child you care for as follows:

[Placeholder]

Section: Further information

What if you have a query or something goes wrong?

If you are unsure about something you can contact the research team for further advice using the contact details at the bottom of this information sheet.

However, if your query has not been handled to your satisfaction, or if you are unhappy and wish to make a formal complaint to someone independent of the research team, then please contact:

[Placeholder]

If you wish to complain or have any concerns about any aspect of the way you have been treated during the course of this study then you should follow the instructions given above.

You can also follow the NHS Complaints Procedure. [Placeholder]

Who should I contact for further information?

If you have any questions or require more information about this study, please contact me using the following contact details:

[Placeholder]

Support and resources

If you feel you would benefit from support, the following organisations may be helpful:

Support for parents and carers:

- Mind: 0300 123 3393 or www.mind.org.uk
- Rethink Mental Illness: 0808 801 0525 or www.rethink.org
- Family Lives (parenting support): 0808 800 2222 or www.familylives.org.uk
- YoungMinds Parent Helpline: 0808 802 5544 or www.youngminds.org.uk

Support for children and young people:

- Childline: 0800 1111 or www.childline.org.uk
- YoungMinds Textline: Text YM to 85258
- The Mix (under 25s): 0808 808 4994 or www.themix.org.uk

Thank you for reading this information sheet and for considering taking part in this research.

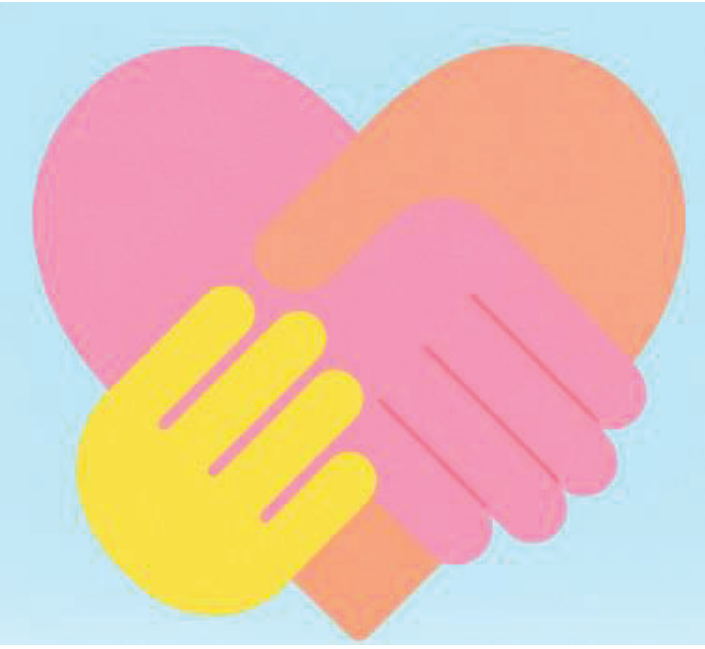

Information Sheet for Children (Ages 8–11)

Study Title: Provision for Parents on Psychiatric Inpatient Units (POPI)

[Staff Introduction Placeholder]

Why are we doing this study?

Sometimes, parents go into hospital because they are not feeling well in their mind. We want to learn what it's like for children when they visit their parent in hospital.

We are trying to find ways to make visits better for children and parents.

What will happen if I say yes?

We'll ask you to talk with someone from our team on a video call.

You will be asked to draw a picture about visiting hospital. You can do this on paper or using a tool on the screen. We will then ask you to describe your picture to us. We will also ask you for ideas about how hospitals can be better for children and young people.

You can have a parent or carer with you if you want.

Do I have to take part?

No. It's your choice. You can say no.

Even if you say yes, you can stop at any time.

You don't have to answer any questions you don't want to.

What will we do with what you tell us?

We'll listen carefully to what you say and use it to help make hospital visits better for other children.

We won't use your name in anything we write down.

We'll keep what you say private unless we are worried that you or someone else is not safe.

We'll also ask if you're happy for us to use your drawing in reports we write about the study.

What if I feel upset?

You can stop the session at any time.

You don’t have to talk about anything you don’t want to.

You can talk to someone you trust, and we’ll give you some ideas for people who can help if needed.

What happens after the chat?

We will send you a small thank-you gift (a £10 voucher).

If you made a digital picture we can send you a copy, if you would like.

What happens to my Information?

We will use your name and contact details to help us do the study, but only a few people will see who you are because your information will be given a special code number instead.

We keep your information safe on secure university computers, only share it with trusted researchers in the UK, and we write about your answers, so no one knows you took part.

You can stop taking part at any time and ask us to delete your information. You can watch a short video that explains how we protect your information and your rights here: https://www.youtube.com/watch?v=VII6V1MgZgY

Who is doing this study?

The study is being run by people at the [Placeholder].

[Placeholder] is in charge. You can ask your parent or carer to contact her if you or they have any questions.

[Placeholder]

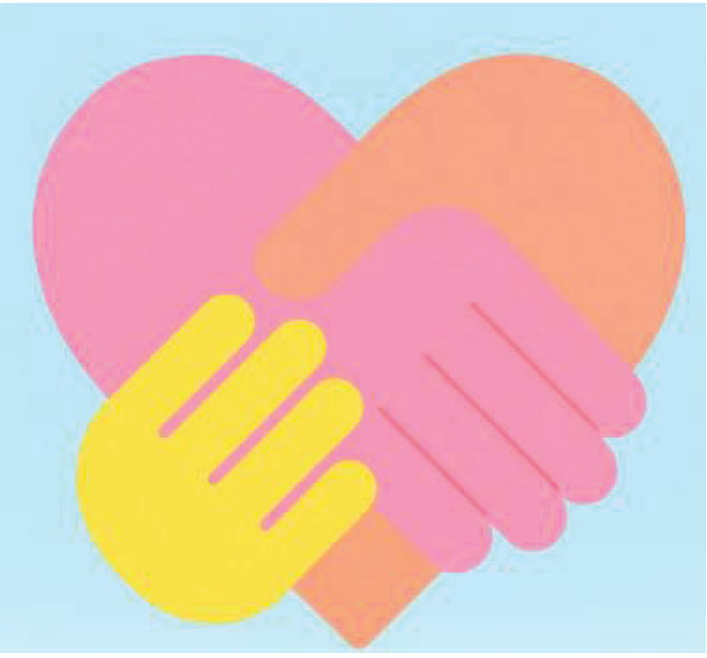

Information Sheet for Children (Ages 12–15)
Study Title: Provision for Parents on Psychiatric Inpatient Units (POPI)

[Staff Introduction Placeholder]

Why are we doing this study?
Sometimes parents go into hospital to get help with their mental health. We want to understand what it's like for children and young people when they visit their parent during this time.

Your experiences can help us make visits better and more supportive for other families in the future.

What will I be asked to do?
You'll be invited to a 30–60 minute chat with someone from the research team on a video call.

You can draw and write about your experience if you like – this is called 'Draw, Write, Tell'. You can do this on paper or using your tablet, phone or computer.

You can have a parent or carer with you if that helps.

Do I have to take part?
No – taking part is your choice. If you say yes, you can still change your mind later.

You can skip any questions you don't want to answer and stop at any time.

Will anyone know what I say?
Only the research team will hear or read what you share.

We will not share your name or anything that could identify you.

Anonymised quotes from your interview may be used in research publications.

If we are worried that you or someone else might not be safe, we may need to tell someone who can help.

At the end of the interview, you will be asked if you're happy for us to use your drawing in any reports we write about the study.

WP1_PIS_CYP_12-15_POPI_

What if talking about this makes me feel upset?
You don't have to talk about anything that makes you uncomfortable.

We can stop the conversation whenever you want.

You can talk to your parent/carer or someone you trust, and we'll also give you information about where to get more support if needed.

Things you can do if you feel upset:

- Talk to someone you trust (like a parent, carer, or friend)
- Do something that makes you feel calm – like drawing, playing, or watching something you like
- Cuddle a pet or soft toy
- Spend time in your favourite place or with your favourite person

If you ever feel sad, worried or just want to talk, here are some people who can help:

Support for children and young people:

- Childline: 0800 1111 or www.childline.org.uk
- YoungMinds Textline: Text YM to 85258
- The Mix (under 25s): 0808 808 4994 or www.themix.org.uk

What happens after the interview?
We'll send you a thank-you gift (a £10 voucher).

If you make a digital drawing, we can give you a copy if you would like one.

Who is running this study?
This research is run by the [Placeholder].

[Placeholder]

What if you have questions or something goes wrong?

If you have questions or are unsure about anything, you can contact the research team using the details at the bottom of this sheet.

If you are not happy with their response, you can contact someone independent at the [Placeholder] to make a formal complaint.

You can also talk to the NHS advice service (PALS), which helps young people and families raise concerns or make complaints. [Placeholder]

Information about your personal data

Who is handling my personal data?

[Placeholder], sponsor of this research and responsible for looking after your information, will act as the 'Data Controller'. The research team will process your personal data on behalf of the controller and is responsible for looking after your information and using it properly. We will use this information as explained in the 'What is the purpose of the study' section above.

What will happen to my personal data?

As a publicly-funded organisation, we have to ensure that when we use **identifiable personal** information from people who have agreed to take part in research, that this data is processed fairly and lawfully. [Placeholder] processes personal data for the purposes of carrying out research in the **public interest** and special category data is processed on an additional condition necessary for **research purposes.** This means that when you agree to take part in this research study, we will use and look after your data in the ways needed to achieve the outcomes of the study.

[Placeholder]

How will we use information about you?

We will need to use information from you for this research project.

This information will include:

- your name
- your contact details.

People will use this information to do the research or to check your records to make sure that the research is being done properly.

People who do not need to know who you are will not be able to see your name or contact details. Your data will have a code number instead.

[Placeholder] is responsible for looking after your information. We will share your information related to this research project with the following types of organisations:

- Regulatory bodies and ethics committees
- Other researchers and institutions (in an anonymised form where you cannot be identified).

We will keep all information about you safe and secure by:

- Anonymising the data
- Keeping any identifiable information in a separate password protected file
- Storing information about you on a secure [Placeholder]-hosted online platform.

Your data will not be shared outside the UK.

How will we use information about you after the study ends?

Once we have finished the study, we will keep some of the data so we can check the results. We will write our reports in a way that no-one can work out that you took part in the study.

We will keep your study data for a maximum of 10 of years. The study data will then be fully anonymised and securely archived or destroyed. Identifiable data will be retained for a maximum of 12 months post-study on a secure university server before being destroyed.

What are your choices about how your information is used?

You can stop being part of the study at any time, without giving a reason, but we will keep information about you that we already have.

You have the right to ask us to access, remove, change or delete data we hold about you for the purposes of the study. You can also object to our processing of your data. We might not always be able to do this if it means we cannot use your data to do the research. If so, we will tell you why we cannot do this.

If you agree to take part in this study, you will have the option to take part in future research using your data saved from this study. To facilitate this, your

anonymised data will be stored in the Figshare data repository where it can be accessed by other researchers in an anonymised form.

Where can you find out more about how your information is used?

You can find out more about how we use your information as follows:

[Placeholder]

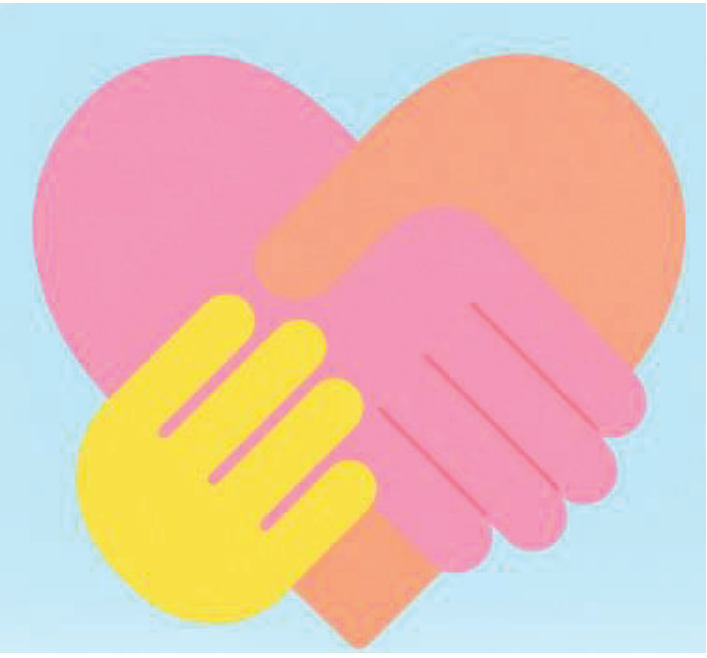

Information Sheet for Young People (Ages 16–18)

Study Title: Provision for Parents on Psychiatric Inpatient Units (POPI)

Why are we doing this study?

Sometimes parents need to spend time in hospital to get support for their mental health. We want to understand what it’s like for young people when they visit their parent during this time. We hope your experiences will help us improve how visits are managed and supported for other families in the future. We are speaking to parents, young people, carers, and healthcare professionals so we can understand different perspectives.

Who is responsible for this study?

[Placeholder of key staff]

This research has been reviewed by an independent group of people, called an Ethics Committee. This study was reviewed and given a favourable opinion by the [Placeholder] which indicates it is ethical, safe and respects the rights of people taking part.

Why have I been asked to take part?

You have been invited because you are aged under 18 and have visited a parent in a mental health hospital at some point between now and July 2021.

What will I be asked to do?

If you take part, you will be invited to a 30–60-minute online conversation (video call) with a member of our research team at a time that works for you.

We are using a 'Draw, Write, Tell' approach – this means you can draw or write about your experiences before talking about them. You can do this on paper or on the screen.

You can have a parent or carer with you during the session if you would like. The conversation will be audio-recorded so we can write it up later.

After the interview we can share your digital drawing with you (if you would like to have it) and we will send you a voucher to thank you for taking part.

After your interview an anonymised (with all identifying data removed) transcript of your interview will be produced (automatic transcription through Microsoft teams, transcription software, and/or transcription by a researcher), after which the audio recording of your interview will be deleted.

Do I have to take part?

No – taking part is your choice. If you say yes, you can still change your mind later without giving a reason.

You can also skip any questions you don't want to answer and stop the interview at any time. If you decide to withdraw, you can ask us to delete your interview up to one month after it takes place, before your information is anonymised.

Are there limits to confidentiality?

Should you disclose information which indicates you or someone else is at risk of harm we may have to contact the appropriate services. In this case we will have to disclose your identity.

Are there any benefits to taking part?

Your experiences will help us understand and improve how visits to parents in hospital are managed and supported. We will give you a £10 voucher to thank you for your time.

Are there any risks?

Some questions may feel personal or bring up difficult memories. You do not have to answer anything that makes you uncomfortable. We can pause or stop the interview at any point. We will give you information about organisations you can contact if you would like support.

How will my information be used?

We will need to use information from you for this research project.

This information will include:

- your name
- your contact details.

People will use this information to do the research or to check your records to make sure that the research is being done properly.

People who do not need to know who you are will not be able to see your name or contact details. Your data will have a code number instead.

[Placeholder] is responsible for looking after your information. We will share your information related to this research project with the following types of organisations:

- Regulatory bodies and ethics committees
- Other researchers and institutions (in an anonymised form where you cannot be identified).

We will keep all information about you safe and secure by:

- Anonymising the data
- Keeping any identifiable information in a separate password protected file
- Storing information about you on a secure [Placeholder]hosted online platform.

Your data will not be shared outside the UK.

<u>How will we use information about you after the study ends?</u>

Once we have finished the study, we will keep some of the data so we can check the results. We will write our reports in a way that no-one can work out that you took part in the study.

We will keep your study data for a maximum of 10 of years. The study data will then be fully anonymised and securely archived or destroyed. Identifiable data will be retained for a maximum of 12 months post-study on a secure university server before being destroyed.

<u>What are your choices about how your information is used?</u>

You can stop being part of the study at any time, without giving a reason, but we will keep information about you that we already have.

You have the right to ask us to access, remove, change or delete data we hold about you for the purposes of the study. You can also object to our processing of your data. We might not always be able to do this if it means we cannot use your data to do the research. If so, we will tell you why we cannot do this.

If you agree to take part in this study, you will have the option to take part in future research using your data saved from this study. To facilitate this, your

anonymised data will be stored in the Figshare data repository where it can be accessed by other researchers in an anonymised form.

Where can you find out more about how your information is used?

You can find out more about how we use your information as follows:

[Placeholder]

What will happen with the results?

We will write a report about what we find and share it on our project website. We may also publish the results in journals and talk about them at conferences, but no one will be able to identify you from this.

What if I have questions or a problem?

If you are unsure about something or have a question, you can contact the research team (details below). If you are unhappy with how you have been treated, you can make a complaint to the [Placeholder]

If you wish to complain or have any concerns about any aspect of the way you have been treated during the course of this study then you should follow the instructions given above.

You can also follow the NHS Complaints Procedure.

[Placeholder]

Contact details

[Placeholder]

Things you can do if you feel upset:

- Talk to someone you trust (like a parent, carer, or friend)
- Do something that makes you feel calm – like drawing, playing, or watching something you like

- Cuddle a pet or soft toy
- Spend time in your favourite place or with your favourite person

If you ever feel sad, worried or just want to talk, here are some people who can help:

Support for children and young people:

- Childline: 0800 1111 or www.childline.org.uk
- YoungMinds Textline: Text YM to 85258
- The Mix (under 25s): 0808 808 4994 or www.themix.org.uk

Participant Consent Form: Parent

Study Title: Mixed methods investigation into provision of family rooms and support for parents admitted to psychiatric inpatient units and their children.

Please initial each box to show your agreement with the item.

1. I confirm that I have read the information sheet [Placeholder] for the above study. I have had the opportunity to consider the information, ask questions and have had these answered satisfactorily. ☐

2. I understand that my participation is voluntary and that I am free to withdraw at any time without giving any reason, without my medical care or legal rights being affected. I understand that if I withdraw after the interview, my data may still be used in anonymised form (where names and identifying details are removed) unless I request otherwise before anonymisation (within one month of the interview). ☐

3. I understand that my interview will be recorded on Microsoft Teams (as video or audio file) depending on my preference of format. ☐

4. I understand that the information I provide will be kept confidential and anonymised in any outputs from the study. ☐

5. I understand that the information collected about me will be used to support other research in the future and may be shared anonymously with other researchers and used in future ethically approved research. ☐

6. I understand that relevant sections of my data collected during the study may be looked at by responsible individuals from [Placeholder], or regulatory authorities. I give permission for these individuals to have access to my data. ☐

7. I understand that anonymised findings from this study may be published or otherwise disseminated. ☐

8. I understand that anonymized direct quotes from my interview may be used in research publications. ☐

9. I understand that my right to anonymity will be waived if I disclose any information which indicates risk of harm to myself or to another person. ☐

10. I understand how the data will be stored and used, and that anonymised data will be retained for up to 10 years in the Figshare data repository. ☐

11. I wish to be contacted to receive findings from this study. ☐

12. I wish to be contacted after the study to be invited to participate in future research. ☐

13. I agree to take part in the above study. ☐

____________________	____________________	____________________
Name of Participant	Date	Electronic signature

Participant Consent Form: Carer

Study Title: Mixed methods investigation into provision of family rooms and support for parents admitted to psychiatric inpatient units and their children.

Please initial each box to show your agreement with the item.

1. I confirm that I have read the information sheet dated 26/01/2026 (version 2.1) for the above study. I have had the opportunity to consider the information, ask questions and have had these answered satisfactorily. ☐

2. I understand that my participation is voluntary and that I am free to withdraw at any time without giving any reason, without my medical care or legal rights being affected. I understand that if I withdraw after the interview, my data may still be used in anonymised form (where names and identifying details are removed) unless I request otherwise before anonymisation (within one month of the interview). ☐

3. I understand that my interview will be recorded on Microsoft Teams (as video or audio file) depending on my preference of format. ☐

4. I understand that the information I provide will be kept confidential and anonymised in any outputs from the study. ☐

5. I understand that the information collected about me will be used to support other research in the future, and may be shared anonymously with other researchers and used in future ethically approved research. ☐

6. I understand that relevant sections of my data collected during the study may be looked at by responsible individuals from [Placeholder], or regulatory authorities. I give permission for these individuals to have access to my data. ☐

☐

7. I understand that anonymised findings from this study may be published or otherwise disseminated.

8. I understand that anonymized direct quotes from my interview may be used in research publications.

9. I understand that my right to anonymity will be waived if I disclose any information which indicates risk of harm to myself or to another person.

10. I understand how the data will be stored and used, and that anonymised data will be retained for up to 10 years in the Figshare data repository.

11. I wish to be contacted to receive findings from this study.

12. I wish to be contacted after the study to be invited to participate in future research.

13. I agree to take part in the above study.

Name of Participant	Date	Electronic signature

Participant Consent Form: Healthcare Professionals

Study Title: Mixed methods investigation into provision of family rooms and support for parents admitted to psychiatric inpatient units and their children.

Please initial each box to show your agreement with the item.

1. I confirm that I have read the information sheet dated [Placeholder] for the above study. I have had the opportunity to consider the information, ask questions and have had these answered satisfactorily. ☐

2. I understand that my participation is voluntary and that I am free to withdraw at any time without giving any reason, without my medical care or legal rights being affected. I understand that if I withdraw after the interview, my data may still be used in anonymised form (where names and identifying details are removed) unless I request otherwise before anonymisation (within one month of the interview). ☐

3. I understand that my interview will be recorded on Microsoft Teams (as video or audio file) depending on my preference of format. ☐

4. I understand that the information I provide will be kept confidential and anonymised in any outputs from the study. ☐

5. I understand that the information collected about me will be used to support other research in the future and may be shared anonymously with other researchers and used in future ethically approved research. ☐

6. I understand that relevant sections of my data collected during the study may be looked at by responsible individuals from [Placeholder], or regulatory authorities. I give permission for these individuals to have access to my data. ☐

7. I understand that anonymised findings from this study may be published or otherwise disseminated. ☐

8. I understand that anonymized direct quotes from my interview may be used in research publications. ☐

9. I understand that my right to anonymity will be waived if I disclose any information which indicates risk of harm to myself or to another person. ☐

10. I understand how the data will be stored and used, and that anonymised data will be retained for up to 10 years in the Figshare data repository. ☐

11. I wish to be contacted to receive findings from this study. ☐

12. I wish to be contacted after the study to be invited to participate in future research. ☐

13. I agree to take part in the above study. ☐

____________________	____________________	____________________
Name of Participant	Date	Electronic signature

Parental Consent Form: Child Interviews
FOR CHILDREN AGED UNDER 16

Study Title: Provision for Parents on Psychiatric Inpatient Units (POPI)

The child (you care for is, referred to as “the child” from this point on) being invited to take part in an interview for a research study. This form is for you, as the parent or legal guardian, to provide consent for their participation. A separate age-appropriate information sheet will be shared with your child.

Please initial each box to show your agreement with the item.

1. I confirm that I have read the information sheet dated [Placeholder] for the above study. I have had the opportunity to consider the information, ask questions and have had these answered satisfactorily. ☐

2. I understand that the child’s participation is voluntary and that they (or I, on their behalf) can withdraw from the study at any time without giving a reason and without any consequences. ☐

3. I understand that if the child chooses to withdraw after the interview, their data may still be used in anonymised form (where names and identifying details are removed) unless I or the child request otherwise before anonymisation (within one month of the interview). ☐

4. I understand that the interview will be recorded on Microsoft Teams (as a video file) ☐

5. I understand that anonymised findings from this study may be published or shared in future research or reports. ☐

6. I understand that relevant sections of data collected during the study may be looked at by responsible individuals from [Placeholder], or regulatory authorities. I give permission for these individuals to have access to the child’s data. ☐

☐

7. I understand that the child's interview will be treated with confidence, and their name or identifying information will not appear in any report or publication.

8. I understand that the information collected about the child I care for will be used in an anonymized form to support other research in the future and may be shared anonymously with other researchers and used in future ethically approved research. ☐

9. I understand that anonymized direct quotes from the interview with the child I care for may be used in research publications. ☐

10. I understand that the right to anonymity will be waived if they disclose information that suggests a risk of harm to themselves or someone else. ☐

11. I understand how the data will be stored and used, and that anonymised data will be retained for up to 10 years in the Figshare data repository. ☐

12. I wish to be contacted to receive findings from this study. ☐

13. I wish to be contacted after the study to be invited to participate in future research. ☐

14. I agree to the child I care for being interviewed for the above study. ☐

Name of child participant: ________________________________

Name of parent/guardian: ________________________________

Relationship to child: ________________________________

Electronic signature of parent/guardian: ______________________________

Date: __________________

Child Assent Form (Ages 8–11)
Study Title: Provision for Parents on Psychiatric Inpatient Units (POPI)

We are doing a study to understand what it's like when a parent stays in a hospital for their mental health. We would like to talk with you about your thoughts and experiences. You don't have to say yes. If you say yes now, you can still change your mind at any time.

Please read each sentence and put your initials (the first letter of your first name and surname) in the box if you agree:

1. I have been told about the study and I understand what it is about. ☐

2. I know that I can ask questions if I want to. ☐

3. I understand that I don't have to take part if I don't want to. ☐

4. I know that I can stop at any time, even if I say yes now. ☐

5. I know that what I say will be kept private unless it raises a worry that I or someone else may not be safe. ☐

6. I know that my interview will be recorded. ☐

7. I am happy to take part in the interview. ☐

Your name: ___________________________________

Your age: __________

Today's date: __________________

Child Assent Form (Ages 12–15)
Study Title: Provision for Parents on Psychiatric Inpatient Units (POPI)

You are being asked if you would like to take part in a research study about what it's like when a parent is in hospital for their mental health. Taking part means talking to a researcher about your thoughts and experiences. You do not have to take part, and you can say no. If you say yes, you can change your mind at any time.

<u>Please read each sentence and put your initials in the box if you agree:</u>

1. I have read or been told about the study and I understand what it is about. ☐

2. I have had the chance to ask questions and they were answered. ☐

3. I understand that I can choose not to take part, and I can stop at any time. ☐

4. I understand that my interview will be recorded and that the words spoken in it will be written down. ☐

5. I understand that what I say will be kept private, unless someone is at risk of harm. ☐

6. I know that the things I say may be written down and used in reports, but my name will not be used. ☐

7. I understand that anonymized direct quotes from my interview may be used in research publications. ☐

8. I am happy to take part in the interview.

Your name: ___________________________________

Your age: __________

Today's date: __________________

Participant Consent Form: CYP 16-18

Study Title: Provision for Parents on Psychiatric Inpatient Units (POPI)

<u>Please initial each box to show your agreement with the item.</u>

1. I confirm that I have read the information sheet dated [Placeholder] for the above study. I have had the opportunity to consider the information, ask questions and have had these answered satisfactorily. ☐

2. I understand that my participation is voluntary and that I am free to withdraw at any time without giving any reason, without my medical care or legal rights being affected. I understand that if I withdraw after the interview, my data may still be used in anonymised form unless I request otherwise before anonymisation (within one month of the interview). ☐

3. I understand that my interview will be recorded on Microsoft Teams (as a video file). ☐

4. I understand that the information I provide will be kept confidential and anonymised in any outputs from the study. ☐

5. I understand that the information collected about me will be used to support other research in the future and may be shared anonymously with other researchers and used in future ethically approved research. ☐

6. I understand that relevant sections of my data collected during the study may be looked at by responsible individuals from the [Placeholder], or regulatory authorities. I give permission for these individuals to have access to my data. ☐

☐

7. I understand that anonymised findings from this study may be published or otherwise disseminated.

8. I understand that anonymized direct quotes from my interview may be used in research publications. ☐

9. I understand that my right to anonymity will be waived if I disclose any information which indicates risk of harm to myself or to another person. ☐

10. I understand how the data will be stored and used, and that anonymised data will be retained for up to 10 years in the Figshare data repository. ☐

11. I wish to be contacted to receive findings from this study. ☐

12. I wish to be contacted after the study to be invited to participate in future research. ☐

13. I agree to take part in the above study. ☐

Name of Participant	Date	Electronic signature

WP1 Topic Guide Parent

1. Introduction

- Briefly explain the study's aim and the reason they have been invited to take part.
- Confirm:
    - Verbal consent
    - Confidentiality:
      "Should you disclose information which indicates you or someone else is at risk of harm we may have to contact the appropriate services. In this case we will have to disclose your identity."
    - Right to stop: Can stop the interview and withdraw from the project at any time.
- Highlight that participant should only provide details of their life which they will feel comfortable having shared.
- Reiterate that participant is welcome to take a break at any time or undertake actions which will make them feel comfortable.

2. Information about the participant

I'm now going to ask some questions about you; this helps us to understand more about you and the other people who are taking part in the study.

- "What is your age?"
- "How would you describe your gender?"
- "How would you describe your ethnicity?"
- *"How long have you lived in the UK?"*
    - *Less than 1 year*
    - *1–5 years*
    - *More than 5 years*
    - *Prefer not to say*

- *Current status in the UK (optional)*
- *"People may have different experiences of living in the UK. Which of the following best describes your current situation?"*
    - *Refugee (granted asylum or humanitarian protection)*
    - *Asylum seeker (waiting for a decision on an asylum claim)*
    - *Other visa or immigration status (e.g. student, work, family)*
    - *British citizen*
    - *Prefer not to say*

- "Which county do you live in?"

- "Which of the following best describes your current financial situation?"
    - I can afford all my basic needs and have money left over for extras
    - I can afford all my basic needs but have little or no money left over
    - I have to cut back on things to afford basic needs
    - I cannot afford my basic needs (e.g., food, housing, utilities)
    - Prefer not to say

- "Please could you share if you have been given a mental health diagnosis? Some people use the names they have been given by services for their conditions, but other people choose names they prefer - for example, voice hearing rather than psychosis."

- "Are you or your family currently supported by a social worker or family professional?

3. Admission context

- "To start, could you tell me a little about your family?"

- "Can you tell me a little about when you were in hospital?"

- "What was it like to be a parent during that time?"

4. Family contact and visits

Aim: to understand how participant experienced family contact

- "Can you share what it was like when your children visited you in hospital?"

- Were there other ways you maintained a connection with your children during this time?

- "Was there any support or information provided to help you or your children with visits or contact?"

- "Was there a specific room or space for visits? What did you think of it?"

5. Improving visit experience

Aim: To explore what supports positive visits:

- “What helped, or would have helped, you and your child feel more in control during visits?”
- “What could make visits more positive or enjoyable for your child?”
- “What helped or would have helped you feel more connected as a family during visits?”
- “Were there any opportunities for social support, for you or your children, during visits?” *(Prompt: e.g., talking with other parents, staff, or peers?)*
- “What could support your wellbeing during or after a family visit?”

6. Suggestions

Aim: generate ideas regarding improvements

- “Looking back, what worked well in terms of support for you as a parent?”
- “What do you think services could improve for parents and families?”
- “What would you want staff to understand about being a parent in hospital?”

7. Ending

- “Is there anything we haven’t talked about that you think is important?

8. Debrief

- Thank the participant for sharing their lived experience which will support this research to make improvements in inpatient care for parents and their children.
- Offer grounding/debrief conversation with lived experience researcher.
- Offer follow-up call and if participant would like to receive one, schedule a time.
- Provide debrief sheet which includes support and discuss.
- Offer if interested to share summary of findings.

WP1 Topic Guide: Carer

1. Introduction

- Briefly explain the study's aim and why they are being interviewed.
- Confirm:
  - Verbal consent
  - Confidentiality:
    "Should you disclose information which indicates you or someone else is at risk of harm we may have to contact the appropriate services. In this case we will have to disclose your identity."
  - Right to stop: Can stop the interview and withdraw from the project at any time.
- Highlight that participant should only provide details of their life which they will feel comfortable having shared.
- Reiterate that participant is welcome to take a break at any time or undertake actions which will make them feel comfortable.

2. Information about the participant

I'm now going to ask some questions about you; this helps us to describe the people who are taking part in the study.

- "What is your age?"
- "How would you describe your gender?"
- "How would you describe your ethnicity?"
- *"How long have you lived in the UK?"*
  - Less than 1 year
  - 1–5 years
  - More than 5 years
  - Prefer not to say
- *Current status in the UK (optional)*
- *"People may have different experiences of living in the UK. Which of the following best describes your current situation?"*
  - Refugee (granted asylum or humanitarian protection)
  - Asylum seeker (waiting for a decision on an asylum claim)
  - Other visa or immigration status (e.g. student, work, family)
  - British citizen
  - Prefer not to say

- “Which county do you live in?”

- “Which of the following best describes your current financial situation?”
    - I can afford all my basic needs and have money left over for extras
    - I can afford all my basic needs but have little or no money left over
    - I have to cut back on things to afford basic needs
    - I cannot afford my basic needs (e.g., food, housing, utilities)
    - Prefer not to say

2. Family context

- “To start, could you tell me a little about your role in the [child name(s*)] life?”
- “Can you tell me about the situation when the [child name(s)] parent was admitted to hospital?”
- “How did you come to be the main carer during that time?”

3. Contact and Visits

Aim: to understand how the child experienced contact with the hospitalised parent

- “Was [child name(s)] able to have contact or visits with their parent during the hospital stay — either through visits, phone/video calls, or messages?”
    - If yes: “How did those visits or contacts go?”
    - If no: “Can you tell me a bit about why contact didn’t happen and how the child responded?”
- “Was any information or support provided to help you or [child name(s)] prepare for visits or contact?”
- “If there were visits, was there a particular space or room used? What was that environment like for the [child name(s)]?”

4. Improving Visit Experience

Aim: To explore what supports positive visits, including how to:

- “What helped, or would have helped, [child name(s)] feel more in control when visiting the hospital?”
- “What helped, or would have helped, keep [child name(s)] calm or positively distracted during the visit?”

- “What helped, or would have helped, [child name(s)] feel connected to their parent during the visit or during the parent’s time in hospital?”

- “Were you or [child name(s)] given any emotional or practical support during this time?”
- “What could support the child’s wellbeing during or after visiting their parent in hospital?”

5. Suggestions

Aim: generate ideas for service improvements

- “What helped most during the time you were caring for [child name(s)]?”
- “What do you think services could improve to better support children and families in this situation?”
- “What would you want staff to understand about children visiting a parent in hospital?”

6. Ending

- “Is there anything we haven’t talked about that you think is important?”

7. Debrief

- Thank the participant for sharing their lived experience which will support this research to make improvements in inpatient care for parents and their children.
- Offer grounding/debrief conversation with lived experience researcher.
- Offer follow-up call and if participant would like to receive one, schedule a time.
- Provide debrief sheet which includes support and discuss.
- Offer if interested to share summary of findings.

WP1 Topic Guide Healthcare Professional

1. Introduction

- Briefly explain the study's aim and the reason they have been invited to take part.
- Stress that it is not an assessment of skills or competencies.
- Confirm:
    - Verbal consent
    - Confidentiality:
      "Should you disclose information which indicates you or someone else is at risk of harm we may have to contact the appropriate services. In this case we will have to disclose your identity."
    - Right to stop: Can stop the interview and withdraw from the project at any time.
- Highlight that participant should only provide details which they will feel comfortable having shared.
- Reiterate that participant is welcome to take a break at any time or undertake actions which will make them feel comfortable.

2. Demographics, Role and Experience

- "To start, could you share your age, gender and ethnicity.
- "Are you able to describe how long you have lived in the UK and your status. If you don't feel comfortable disclosing this we can move on to the next question"
- "Which county do you live in?"
- "Now, please can you briefly describe your role and the kind of work you do/did on the ward?"
- "How often do/did you work with patients who are parents?"

3. Family Contact and Visits

Aim: to understand how healthcare professionals experience and support family contact

- "How are/were visits from children or family members usually managed on your ward?"
- "What tends/tended to work well when arranging or supporting visits from children?"
- "What are some of the challenges that come/came up around family visits?"
- "Is/was there a specific room or space used for family visits? What do you think about it?"
- "What kinds of support or resources are currently available to help with family visits?"

4. Improving Visit Experience

Aim: to explore what supports positive visits

- "What would help parents and children feel more in control during visits?"
- "What would help make visits more positive or enjoyable for children?"
- "What would support families to feel more connected during visits?"
- "What would support the wellbeing of parents during and after visits?"

5. Suggestions

Aim: generate ideas regarding improvements

- "What would help staff feel more confident or supported in working with parents and children?"
- "What would make it easier to facilitate parent/child contacts?"

6. Ending

- "Is there anything we haven't talked about that you think is important?"

7. Debrief

- Thank the participant for sharing their experience which will support this research to make improvements in inpatient care for parents and their children.
- Offer grounding/debrief conversation with lived experience researcher.
- Offer follow-up call and if participant would like to receive one, schedule a time.
- Provide debrief sheet which includes support and discuss.
- Offer if interested to share summary of findings.

WP1 Topic Guide CYP

1. Introduction

Explain the study's aim in simple terms:

- "We're doing a project to learn about what it's like for children and young people when a parent is in hospital. You've been invited because you visited your parent in hospital, and we'd really like to hear about your experiences."

Reassure about confidentiality and consent:

- "You can choose what you want to tell me. You don't have to answer anything you don't want to. You can stop at any time, or ask to take a break."
- We will keep what you tell us private and only use the information in ways which mean you cannot be identified. If you tell us something which worries us that you or someone else is not safe, we may have to share it.

Emphasise safety and comfort:

- "If there's anything you don't want to talk about, that's absolutely fine. You can speak to your [mum/dad/carer type] at any point during our session together."

2. Information about the CYP

To start with I'm now going to ask a few questions about you; this helps us to understand who is taking part.

- "What is your age?"
- A) "How would you describe your gender?" (11 onwards)
- B) "Do you think of yourself as a boy, a girl, or something else?" (8-10)
- A) "Which county do you live in?" (11 onwards)
- B) Do you know the name of the village, town or city where you live? (8-10)

3. Getting started: drawing activity

Introduce the activity:

- "To help you tell me about your experience, I am going to ask you to start with a drawing – this can also include writing if you would like. You can use paper and show it on camera, use the whiteboard here, or draw using [THE APP], whichever you prefer."

If using the online tool do a practice exercise to help the CYP familiarise with the tool.

Invite the drawing:

- "Can you think back to a time when you visited your mum/dad/carer in hospital. You can draw whatever comes to mind when you think about that time. It could be about the place, how you felt, or anything else. "

  "If you want to you can use words and writing as well."

3. Telling the story: interpreting the drawing

Aim: Open ended questions to help the CYP describe their drawing and experience

- "Can you tell me about what you've drawn?"
- "What is happening in the picture?"
- "Is there anything written or shown that you'd like to explain?"
- "How were you feeling in that moment?"

4. Exploring the visit experience

Aim: Understand how CYP experienced the visit

- "What was it like visiting your parent in hospital?"
- "Was there anything you liked or didn't like?"
- "Was there a special room or place where the visit happened? What was it like?"
- "Did anyone talk to you before or after the visit about what would happen?"
- "Did anything or anyone help you feel better when you visited hospital?"

5. Improving Visits

Aim: Explore what would help CYP feel safe, connected, and supported

Intro: "Sometimes visits to hospital can be a bit tricky. We want to understand what could make things better."

- "Is there anything that you would like to have known about before you visited your mum/dad/carer for the first time to help you feel prepared?
- "Is there anything that would have helped make visits more fun or enjoyable?"
- "Can you think of any things that would have helped you feel close to your mum/dad/carer when you visited them?"

- "Did anyone else help you during the visit — like the doctors and nurses, family, or other children?"
- "Is there anything that you can think of that would have been helpful after you visited the hospital?

6. Ending and follow-up

- "Is there anything else you want to say about visiting your mum/dad/carer?"

7. Debrief

- Thank them for sharing their experience.
- Offer a follow-up call to them/their parent/carer.
- Offer to email their drawing to them or their carer if they'd like?
- Provide debrief sheet which includes support and discuss.
- Explain what will happen next in the project including how they can access an age appropriate report.

PARTICIPANT INFORMATION SHEET NHS

Title of Study: Mixed methods investigation into provision of family rooms and support for parents admitted to psychiatric inpatient units and their children.

PLEASE KEEP A COPY OF THIS INFORMATION SHEET FOR YOUR RECORDS

Section: Taking Part

Invitation Paragraph

We would like to invite you to participate in this research project. You should only participate if you want to; choosing not to take part will not disadvantage you in any way. Before you decide whether you want to take part, it is important for you to understand why the research is being done and what your participation will involve. Please discuss the study with others if you wish. If you have any questions you can contact us using the contact details at the end of this information sheet.

What is the purpose of the study?

The aim of this study is to understand the experience of family visits when a parent is treated in a mental health inpatient hospital.
We are specifically interested in how we can improve the experience of these visits for parents and their children.
We are inviting **parents**, **carers of children** who visited a parent in hospital and **healthcare professionals** to answer a brief questionnaire.

Who is responsible for this study?

[Placeholder]

Why have I been invited to take part?
You are invited to participate in this study because you are a:

A parent (of a child 18 or under) and you are currently staying on a mental health inpatient unit or have stayed on one at some point since July 2021.

A carer of a child who visited their parent in a mental health hospital

A healthcare professional who has worked in an adult mental health inpatient unit in the last two years for a minimum of six months.

Do I have to take part?

Participation is voluntary and you do not have to take part. You can stop at any time simply by exiting the questionnaire. Please contact us if there is anything that is not clear, if you have any questions, or if you would like more information.

What will happen to me if I decide to take part?

If you decide to take part, you will need to:

1. Read this online information sheet
2. Confirm that you meet the eligibility criteria
3. Provide online consent
4. Complete a short questionnaire (approx. 10–15 minutes)
5. Receive debrief information, which includes what happens next in the project along with signposting to support resources, at the end.

You will not be asked for any identifying information unless you choose to enter a prize draw.

What happens if I do not want to take part or if I change my mind?

You can stop completing the questionnaire at any time by closing your browser. Because the questionnaire is anonymous, it will not be possible to remove your responses once submitted.

What are the possible benefits in taking part?

While there is no direct benefit, your responses will help us improve how inpatient mental health wards support families. You can also choose to enter a prize draw to win a £50 shopping voucher.

What are the possible risks or benefits?

A possible disadvantage to taking part in the study is that some interview questions may prompt reflection on difficult experiences. In order to reduce any potential risks, you will not be required to answer any question you find uncomfortable. Debriefing information and signposting to support resources will be provided.

How is the project being funded?

[Placeholder]

What will happen to the results of the study?

We will produce a final report summarising the main findings. This will be available on our project website [Placeholder].
This research may be published in peer reviewed academic journals under open access conditions. This means the article can be read for free online by anyone who wishes to. The findings will be presented to stakeholder groups and at conferences.

You will be sent a summary of the results of the study if you indicate you would like to receive one.

Who has reviewed this study?

This research has been reviewed by an independent group of people, called an Ethics Committee. This study was reviewed and given a favourable opinion by the [Placeholder].

Section: Your personal data

How will we use information about you?

We will need to use information from you for this research project.

This information will include:

- your name
- your contact details.

People will use this information to do the research or to check your records to make sure that the research is being done properly.

People who do not need to know who you are will not be able to see your name or contact details. Your data will have a code number instead.

[Placeholder] is responsible for looking after your information. We will share your information related to this research project with the following types of organisations:

- Regulatory bodies and ethics committees
- Other researchers and institutions (in an anonymised form where you cannot be identified).

We will keep all information about you safe and secure by:

- Anonymising the data
- Keeping any identifiable information in a separate password protected file
- Storing information about you on a secure [Placeholder] -hosted online platform.

Your data will not be shared outside the UK.

How will we use information about you after the study ends?

Once we have finished the study, we will keep some of the data so we can check the results. We will write our reports in a way that no-one can work out that you took part in the study.

We will keep your study data for a maximum of 10 of years. The study data will then be fully anonymised and securely archived or destroyed. Identifiable data will be retained for a maximum of 12 months post-study on a secure university server before being destroyed.

What are your choices about how your information is used?

- You can stop being part of the study at any time, without giving a reason, but we will keep information about you that we already have
- You have the right to ask us to access, remove, change or delete data we hold about you for the purposes of the study. You can also object to our processing of your data. We might not always be able to do this if it means we cannot use your data to do the research. If so, we will tell you why we cannot do this.
- If you agree to take part in this study, you will have the option to take part in future research using your data saved from this study. To facilitate this, your anonymised data will be stored in the Figshare data repository where it can be accessed by other researchers in an anonymised form.

Where can you find out more about how your information is used?

You can find out more about how we use your information as follows:

[Placeholder]

Section: Further information

What if you have a query or something goes wrong?

If you are unsure about something you can contact the research team for further advice using the contact details at the bottom of this information sheet.

However, if your query has not been handled to your satisfaction, or if you are unhappy and wish to make a formal complaint to someone independent of the research team, then please contact:

[Placeholder]

If you wish to complain or have any concerns about any aspect of the way you have been treated during the course of this study then you should follow the instructions given above.

You can also follow the NHS Complaints Procedure. [Placeholder]

Who should I contact for further information?

If you have any questions or require more information about this study, please contact me using the following contact details:

[Placeholder]

Support if you're feeling upset or distressed

We know that thinking about difficult experiences can sometimes create upsetting feelings. To help you look after yourself, we suggest you please consider making use of any things you usually find helpful. Suggestions from previous participants include:

- Contacting a friend, family member or support worker afterwards
- Having your pet nearby
- Using quiet fidget toys or stimming aids
- Spending time in a comforting space or doing an activity that soothes you (e.g., music, a walk, favourite TV show)

If you feel you would benefit from additional support, the following organisations may be helpful:

- Mind: 0300 123 3393 or www.mind.org.uk
- Rethink Mental Illness: 0808 801 0525 or www.rethink.org
- Family Lives (parenting support): 0808 800 2222 or www.familylives.org.uk
- YoungMinds Parent Helpline: 0808 802 5544 or www.youngminds.org.uk

Thank you for reading this information sheet and for considering taking part in this research.

PARTICIPANT INFORMATION SHEET NHS (Paper)

Title of Study: Mixed methods investigation into provision of family rooms and support for parents admitted to psychiatric inpatient units and their children.

PLEASE KEEP A COPY OF THIS INFORMATION SHEET FOR YOUR RECORDS

Section: Taking Part

Invitation Paragraph

We would like to invite you to participate in this research project. You should only participate if you want to; choosing not to take part will not disadvantage you in any way. Before you decide whether you want to take part, it is important for you to understand why the research is being done and what your participation will involve. Please discuss the study with others if you wish. If you have any questions you can contact us using the contact details at the end of this information sheet.

What is the purpose of the study?

The aim of this study is to understand the experience of family visits when a parent is treated in a mental health inpatient hospital.
We are specifically interested in how we can improve the experience of these visits for parents and their children.
We are inviting **parents**, **carers of children** who visited a parent in hospital and **healthcare professionals** to answer a brief questionnaire.

Who is responsible for this study?

[Placeholder]

Why have I been invited to take part?

You are invited to participate in this study because you are a:

A parent (of a child 18 or under) and you are currently staying on a mental health inpatient unit or have stayed on one at some point since July 2021.

A carer of a child who visited their parent in a mental health hospital.

A healthcare professional who has worked in an adult mental health inpatient unit in the last two years for a minimum of six months.

Do I have to take part?

Participation is voluntary and you do not have to take part. At any time you can stop filling out the questionnaire and discarding it. Please contact us or ask staff if there is anything that is not clear, if you have any questions, or if you would like more information.

What will happen to me if I decide to take part?

If you decide to take part, you will need to:

1. Read this information sheet
2. Confirm that you meet the eligibility criteria
3. Complete the consent form
4. Complete the short questionnaire (approx. 10–15 minutes)
5. Receive debrief information, which includes what happens next in the project along with signposting to support resources, which are contained on the final pages of the questionnaire.

The member of the research or clinical team who provided you with this questionnaire pack will send a scanned version of it to the research team after which time they will securely destroy the paper version.

You will not be asked for any identifying information unless you choose to enter a prize draw.

What happens if I do not want to take part or if I change my mind?

You can stop completing the questionnaire at any time and discarding of the questionnaire yourself or asking staff to discard of it. Because the questionnaire is anonymous, it will not be possible to remove your responses once they have been shared with the study team.

What are the possible benefits in taking part?

While there is no direct benefit, your responses will help us improve how inpatient mental health wards support families. You can also choose to enter a prize draw to win one of four £50 shopping voucher.

What are the possible risks or benefits?

A possible disadvantage to taking part in the study is that some interview questions may prompt reflection on difficult experiences. In order to reduce any potential risks, you will not be required to answer any question you find uncomfortable. Debriefing information and signposting to support resources will be provided.

How is the project being funded?

[Placeholder]

What will happen to the results of the study?

We will produce a final report summarising the main findings. This will be available on our project website inpatientfamilies.org
This research may be published in peer reviewed academic journals under open access conditions. This means the article can be read for free online by anyone who wishes to. The findings will be presented to stakeholder groups and at conferences.

Who has reviewed this study?

This research has been reviewed by an independent group of people, called an Ethics Committee. This study was reviewed and given a favourable opinion by [Placeholder].

Section: Your personal data

How will we use information about you?
We will need to use information from you for this research project.

This information will include:
- your name
- your contact details.

People will use this information to do the research or to check your records to make sure that the research is being done properly.

People who do not need to know who you are will not be able to see your name or contact details. Your data will have a code number instead.

The [Placeholder] is responsible for looking after your information. We will share your information related to this research project with the following types of organisations:
- Regulatory bodies and ethics committees
- Other researchers and institutions (in an anonymised form where you cannot be identified).

We will keep all information about you safe and secure by:
- Anonymising the data
- Keeping any identifiable information in a separate password protected file

Storing information about you on a secure [Placeholder]-hosted online platform.

Your data will not be shared outside the UK.

How will we use information about you after the study ends?

Once we have finished the study, we will keep some of the data so we can check the results. We will write our reports in a way that no-one can work out that you took part in the study.
We will keep your study data for a maximum of 10 of years. The study data will then be fully anonymised and securely archived or destroyed. Identifiable data will be retained for a maximum of 12 months post-study on a secure university server before being destroyed.

What are your choices about how your information is used?

- You can stop being part of the study at any time, without giving a reason
- You have the right to ask us to access, remove, change or delete data we hold about you for the purposes of the study. You can also object to our processing of your data. We might not always be able to do this if it means we cannot use your data to do the research. If so, we will tell you why we cannot do this.
- If you agree to take part in this study, you will have the option to take part in future research using your data saved from this study. To facilitate this, your anonymised data will be stored in the Figshare data repository where it can be accessed by other researchers in an anonymised form.

Where can you find out more about how your information is used?
You can find out more about how we use your information as follows:

[Placeholder]

Section: Further information

What if you have a query or something goes wrong?

If you are unsure about something you can contact the research team for further advice using the contact details at the bottom of this information sheet.

However, if your query has not been handled to your satisfaction, or if you are unhappy and wish to make a formal complaint to someone independent of the research team, then please contact:

[Placeholder]

If you wish to complain or have any concerns about any aspect of the way you have been treated during the course of this study then you should follow the instructions given above.

You can also follow the NHS Complaints Procedure. [Placeholder]

Who should I contact for further information?

If you have any questions or require more information about this study, please contact me using the following contact details:

[Placeholder]

<u>Support if you're feeling upset or distressed</u>

We know that thinking about difficult experiences can sometimes create upsetting feelings. To help you look after yourself, we suggest you please consider making use of any things you usually find helpful. Suggestions from previous participants include:

- Contacting a friend, family member or support worker afterwards
- Having your pet nearby
- Using quiet fidget toys or stimming aids
- Spending time in a comforting space or doing an activity that soothes you (e.g., music, a walk, favourite TV show)

If you feel you would benefit from additional support, the following organisations may be helpful:

- Mind: 0300 123 3393 or www.mind.org.uk
- Rethink Mental Illness: 0808 801 0525 or www.rethink.org
- Family Lives (parenting support): 0808 800 2222 or www.familylives.org.uk
- YoungMinds Parent Helpline: 0808 802 5544 or www.youngminds.org.uk

Thank you for reading this information sheet and for considering taking part in this research.

Consent Form – Work Package 2

Study Title: Provision for Parents on Psychiatric Inpatient Units (POPI)

[Placeholder]

Please read the following statement, initial each box to show your agreement, and confirm your consent by checking the box at the end.

<u>Please tick the box to confirm,</u>

1. I confirm that I have read the information sheet dated [Placehodler] for the above study. I have had the opportunity to consider the information, ask questions and have had these answered satisfactorily. ☐

2. I understand that my participation is voluntary and that I am free to stop completing the questionnaire at any time before submission without giving a reason. ☐

3. I understand that any information I provide will be anonymous (where names and identifying details are removed) and kept confidential. ☐

4. I understand that my anonymous data may be used in future ethically approved research. ☐

5. I understand that anonymized direct quotes may be used in research publications. ☐

6. I understand that anonymised data may be used in research reports, presentations, and academic publications. ☐

7. I confirm that I agree to take part in this study. ☐

WP2 Questionnaire Parent

Section 1: About You

1. What is your age? (in years): _________

2. How would you describe your gender?

- ☐ Male
- ☐ Female
- ☐ Non-binary
- ☐ Prefer to self-describe: _______________
- ☐ Prefer not to say

3. How would you describe your ethnicity?

- ☐ White British
- ☐ White other
- ☐ Black/Black British
- ☐ Asian/Asian British
- ☐ Mixed/multiple ethnic groups
- ☐ Other ethnic group: _________________
- ☐ Prefer not to say

4. “How long have you lived in the UK?”

- ☐ Less than 1 year
- ☐ 1–5 years
- ☐ More than 5 years
- ☐ Prefer not to say

5. Current status in the UK (optional)

People may have different experiences of living in the UK. Which of the following best describes your current situation?

- ☐ Refugee (granted asylum or humanitarian protection)
- ☐ Asylum seeker (waiting for a decision on an asylum claim)
- ☐ Other visa or immigration status (e.g. student, work, family)
- ☐ British citizen
- ☐ Prefer not to say

6. Which county do you live in?: _________________________________

7. Which of the following best describes your current financial situation?

☐ I can afford all my basic needs and have money left over for extras
☐ I can afford all my basic needs but have little or no money left over
☐ I have to cut back on things to afford basic needs
☐ I cannot afford my basic needs (e.g., food, housing, utilities)
☐ Prefer not to say

8. Please could you share if you have been given a mental health diagnosis? [Some people use the names they have been given by services for their conditions but other people choose names they prefer, for example voice hearing rather than psychosis]:

____

____

____

9. How many children do you have: ___________________

10. What is the age of your child / are the ages of your oldest and youngest child? [years and months]:

____

____

11. Are you or your family currently supported by a social worker or family professional?

☐ Yes
☐ No
☐ Not sure
☐ Prefer not to say

Section 2: Your Hospital Stay

12. In what year were you most recently admitted to a hospital for your mental health?:

____

13. Were you admitted voluntarily or under section?

☐ Voluntarily
☐ Under section
☐ Not sure / don't remember
☐ Prefer not to say

14. Did your child(ren) visit you in hospital?

☐ Yes, they visited
☐ No, but I wanted them to
☐ No, and I did not want them to

15. If your child(ren) visited: Where did the visit(s) take place?

- ☐ Family room
- ☐ Bedroom or ward area
- ☐ Garden or outdoor space
- ☐ Other (please describe):

____

[**Online Only:** question only shown if question 14 answered "Yes, they visited"]

16. How did the space where the visit took place make you feel?

Very unhappy	☐	☐	☐	☐	☐	Very happy
Relaxed	☐	☐	☐	☐	☐	Stressed
Calm	☐	☐	☐	☐	☐	Anxious
Safe	☐	☐	☐	☐	☐	Unsafe
Bored	☐	☐	☐	☐	☐	Interested
Tired	☐	☐	☐	☐	☐	Energised
Confused	☐	☐	☐	☐	☐	In control
Connected to others	☐	☐	☐	☐	☐	Not connected

[**Online Only:** question only shown if question 14 answered "Yes, they visited", this question will be shown with as 5 scale-point sliding scales.]

17. What would help reduce stress for parents or children during a visit?

____

____

____

____

Section 3: Potential features of family visits?

18. Below are features which parents have identified as being important when their child visits them in hospital. For each one, please tick how you think it would make you feel (select all that apply).

[**Online Only:** For items for which participant selected 'Other feeling (please specify)' participants will be presented with an open text box to specify]

	Happy	Relaxed	Calm	Safe	Interested	Energised	In control	Connected to others	No different	It would make things worse	Other feeling (please specify)
Information about how to arrange/book visits	☐	☐	☐	☐	☐	☐	☐	☐	☐	☐	
A confirmation message of visit details (e.g., by email or text) before visits	☐	☐	☐	☐	☐	☐	☐	☐	☐	☐	
Information for my family before the visit (e.g., what the hospital visiting room is like, rules about bringing items in)	☐	☐	☐	☐	☐	☐	☐	☐	☐	☐	
Opportunity to plan a visit with staff beforehand	☐	☐	☐	☐	☐	☐	☐	☐	☐	☐	
Signs around the hospital to help my family find their way around	☐	☐	☐	☐	☐	☐	☐	☐	☐	☐	
A named staff member to support me during visits	☐	☐	☐	☐	☐	☐	☐	☐	☐	☐	
Staff available nearby during visits if needed	☐	☐	☐	☐	☐	☐	☐	☐	☐	☐	
Staff present in the room during visits	☐	☐	☐	☐	☐	☐	☐	☐	☐	☐	

	Happy	Relaxed	Calm	Safe	Interested	Energised	In control	Connected to others	No different	It would make things worse	Other feeling (please specify)
A dedicated room/space for family visits.	☐	☐	☐	☐	☐	☐	☐	☐	☐	☐	
Visits being private (visitors not visible to ward or other patients)	☐	☐	☐	☐	☐	☐	☐	☐	☐	☐	
Access to outdoor visiting space	☐	☐	☐	☐	☐	☐	☐	☐	☐	☐	
Comfortable soft furnishing in the family visit room (e.g. fabric sofa)	☐	☐	☐	☐	☐	☐	☐	☐	☐	☐	
A room set-up that could be rearranged for different activities (e.g. playing on floor)	☐	☐	☐	☐	☐	☐	☐	☐	☐	☐	
Activities such as books, games or toys in the family visit room	☐	☐	☐	☐	☐	☐	☐	☐	☐	☐	
Access to technology accessories (e.g. plugs, WIFI)	☐	☐	☐	☐	☐	☐	☐	☐	☐	☐	
Access to a TV	☐	☐	☐	☐	☐	☐	☐	☐	☐	☐	
A colourful family room with artworks or decorations	☐	☐	☐	☐	☐	☐	☐	☐	☐	☐	
Access to drinks or snacks during visits	☐	☐	☐	☐	☐	☐	☐	☐	☐	☐	
A check-in with staff after the visit	☐	☐	☐	☐	☐	☐	☐	☐	☐	☐	

Section 4: Prioritising Improvements

19. Please select the FIVE elements which feel most important to you.

[**Online Only:** Participants will be presented with the list of items from the matrix (section 3). that were not rated 'It would make things worse']

	Select the five items which are most important	Rank the five selected items most important (one) to least important (five)
Information about how to arrange/book visits	☐	
A confirmation message of visit details (e.g., by email or text) before visits	☐	
Information for my family before the visit (e.g., what the hospital visiting room is like, rules about bringing items in)	☐	
Opportunity to plan a visit with staff beforehand	☐	
Signs around the hospital to help my family find their way around	☐	
A named staff member to support me during visits	☐	
Staff available nearby during visits if needed	☐	
Staff present in the room during visits	☐	
A dedicated room/space for family visits.	☐	
Visits being private (visitors not visible to ward or other patients)	☐	
Access to outdoor visiting space	☐	
Comfortable soft furnishing in the family visit room (e.g. fabric sofa)	☐	
A room set-up that could be rearranged for different activities (e.g. playing on floor)	☐	
Activities such as books, games or toys in the family visit room	☐	
Access to technology accessories (e.g. plugs, WIFI)	☐	
Access to a TV	☐	
A colourful family room with artworks or decorations	☐	
Access to drinks or snacks during visits	☐	
A check-in with staff after the visit	☐	

20. Please now rank those five elements from most important (one) to least important (five).

[**Online Only:** We will display the five elements selected by the parent in question 19]

Final Comments

21. Is there anything else you would like to share about your experience or that you think would improve family visits for other parents?

____

____

WP2 Questionnaire: Carer

Section 1: About You

1. What is your age? (in years)?: _________

2. How would you describe your gender?

☐ Male
☐ Female
☐ Non-binary
☐ Prefer to self-describe: _______________
☐ Prefer not to say

3. How would you describe your ethnicity?

☐ White British
☐ White other
☐ Black/Black British
☐ Asian/Asian British
☐ Mixed/multiple ethnic groups
☐ Other ethnic group: _________________
☐ Prefer not to say

4. How long have you lived in the UK?

☐ Less than 1 year
☐ 1–5 years
☐ More than 5 years
☐ Prefer not to say

5. Current status in the UK (optional)

People may have different experiences of living in the UK. Which of the following best describes your current situation?

☐ Refugee (granted asylum or humanitarian protection)
☐ Asylum seeker (waiting for a decision on an asylum claim)
☐ Other visa or immigration status (e.g. student, work, family)
☐ British citizen
☐ Prefer not to say

6. Which county do you live in?:________________________________

7. What is your relationship to the child(ren)?

- ☐ Parent
- ☐ Step-parent
- ☐ Grandparent
- ☐ Foster carer
- ☐ Family friend
- ☐ Other (please describe): ______

8. How old was the child/the oldest and youngest children at the time of the hospital admission? [years and months]

____

____

9. Were you living with the child at the time of the parent's hospital admission?

- ☐ Yes
- ☐ No
- ☐ Prefer not to say

10. Was a social worker or family professional supporting the child(ren) during this time?

- ☐ Yes
- ☐ No
- ☐ Not sure
- ☐ Prefer not to say

Section 2: The Parent's Hospital Stay

11. When was the parent admitted to hospital for their mental health? (approximate date)

____

12. Did the child(ren) visit the parent in hospital?

- ☐ Yes
- ☐ No

13. If no, why not?

[**Online Only:** question only shown if question 12 answered "Yes"]

____

____

14. If yes: How many times did they visit?: ______________________

[**Online Only:** question only shown if question 12 answered "Yes"]

15. Where did the visit(s) take place?

[**Online Only:** question only shown if question 12 answered "Yes"]

- ☐ Family room
- ☐ Ward area
- ☐ Garden or outdoor space
- ☐ Other (please describe): ______

16. Who accompanied the child during the visit?

[**Online Only:** question only shown if question 12 answered "Yes"]

____

Section 3: The experience of visiting

17. How do you think the child felt in the visiting space?

Very unhappy	☐	☐	☐	☐	☐	Very happy
Relaxed	☐	☐	☐	☐	☐	Stressed
Calm	☐	☐	☐	☐	☐	Anxious
Safe	☐	☐	☐	☐	☐	Unsafe
Bored	☐	☐	☐	☐	☐	Interested
Tired	☐	☐	☐	☐	☐	Energised
Confused	☐	☐	☐	☐	☐	In control
Connected to others	☐	☐	☐	☐	☐	Not connected

[**Online Only:** question only shown if question 12 answered "Yes, they visited", this question will be shown with as 5 scale-point sliding scales.]

18. What do you think would help reduce stress for children during a visit?

Section 4: Potential features of family visits?

19. Below are features which parents and carers have identified as being important when a child visits a parent in hospital. For each one, please tick how you think it would make **the child** feel (select all that apply).

[**Online Only:** For items for which participant selected 'Other feeling (please specify)' participants will be presented with an open text box to specify]

	Happy	Relaxed	Calm	Safe	Interested	Energised	In control	Connected to others	No different	It would make things worse	Other feeling (please specify)
**Information about how to arrange/book visits**	☐	☐	☐	☐	☐	☐	☐	☐	☐	☐	
**A confirmation message of visit details (e.g., by email or text) before visits**	☐	☐	☐	☐	☐	☐	☐	☐	☐	☐	
**Information for them before the visit (e.g., what the hospital visiting room is like, rules about bringing items in)**	☐	☐	☐	☐	☐	☐	☐	☐	☐	☐	
**Signs around the hospital to help them find their way around**	☐	☐	☐	☐	☐	☐	☐	☐	☐	☐	
**A named staff member to support during visits**	☐	☐	☐	☐	☐	☐	☐	☐	☐	☐	
**Staff available nearby during visits if needed**	☐	☐	☐	☐	☐	☐	☐	☐	☐	☐	
**Staff present in the room during visits**	☐	☐	☐	☐	☐	☐	☐	☐	☐	☐	
**A dedicated room/space for family visits.**	☐	☐	☐	☐	☐	☐	☐	☐	☐	☐	

	Happy	Relaxed	Calm	Safe	Interested	Energised	In control	Connected to others	No different	It would make things worse	Other feeling (please specify)
**Visits being private (visitors not visible to ward or other patients)**	☐	☐	☐	☐	☐	☐	☐	☐	☐	☐	
**Access to outdoor visiting space**	☐	☐	☐	☐	☐	☐	☐	☐	☐	☐	
**Comfortable soft furnishing in the family visit room (e.g. fabric sofa)**	☐	☐	☐	☐	☐	☐	☐	☐	☐	☐	
**A room set-up that could be rearranged for different activities (e.g. playing on floor)**	☐	☐	☐	☐	☐	☐	☐	☐	☐	☐	
**Activities such as books, games or toys in the family visit room**	☐	☐	☐	☐	☐	☐	☐	☐	☐	☐	
**Access to technology accessories (e.g. plugs, WIFI)**	☐	☐	☐	☐	☐	☐	☐	☐	☐	☐	
**Access to a TV**	☐	☐	☐	☐	☐	☐	☐	☐	☐	☐	
**A colourful family room with artworks or decorations**	☐	☐	☐	☐	☐	☐	☐	☐	☐	☐	
**Access to drinks or snacks during visits**	☐	☐	☐	☐	☐	☐	☐	☐	☐	☐	
**A check-in with staff after the visit**	☐	☐	☐	☐	☐	☐	☐	☐	☐	☐	

20. Below are features which parents and carers have identified as being important when a child visits their parent in hospital. For each one, please tick how you think it would make **you** feel (select all that apply).

[**Online Only:** For items for which participant selected 'Other feeling (please specify)' participants will be presented with an open text box to specify]

	Happy	Relaxed	Calm	Safe	Interested	Energised	In control	Connected to others	No different	It would make things worse	Other feeling (please specify)
Information about how to arrange/book visits	☐	☐	☐	☐	☐	☐	☐	☐	☐	☐	
A confirmation message of visit details (e.g., by email or text) before visits	☐	☐	☐	☐	☐	☐	☐	☐	☐	☐	
Information for my family before the visit (e.g., what the hospital visiting room is like, rules about bringing items in)	☐	☐	☐	☐	☐	☐	☐	☐	☐	☐	
Signs around the hospital to help my family find their way around	☐	☐	☐	☐	☐	☐	☐	☐	☐	☐	
A named staff member to support me during visits	☐	☐	☐	☐	☐	☐	☐	☐	☐	☐	
Staff available nearby during visits if needed	☐	☐	☐	☐	☐	☐	☐	☐	☐	☐	
Staff present in the room during visits	☐	☐	☐	☐	☐	☐	☐	☐	☐	☐	
A dedicated room/space for family visits.	☐	☐	☐	☐	☐	☐	☐	☐	☐	☐	
Visits being private (visitors not visible to ward or other patients)	☐	☐	☐	☐	☐	☐	☐	☐	☐	☐	
Access to outdoor visiting space	☐	☐	☐	☐	☐	☐	☐	☐	☐	☐	
Access to drinks or snacks during visits	☐	☐	☐	☐	☐	☐	☐	☐	☐	☐	
A check-in with staff after the visit	☐	☐	☐	☐	☐	☐	☐	☐	☐	☐	

Section 5: Prioritising Improvements

21. Please select the FIVE elements which feel most important to you and the child.

[**Online Only:** Participants will be presented with the list of items from the matrix (question 19). that were not rated 'It would make things worse']

	Select the five items which are most important	Rank the selected items most important (one) to least important (five)
**Information about how to arrange/book visits**	☐	
**A confirmation message of visit details (e.g., by email or text) before visits**	☐	
**Information for them before the visit (e.g., what the hospital visiting room is like, rules about bringing items in)**	☐	
**Signs around the hospital to help them find their way around**	☐	
**A named staff member to support during visits**	☐	
**Staff available nearby during visits if needed**	☐	
**Staff present in the room during visits**	☐	
**A dedicated room/space for family visits.**	☐	
**Visits being private (visitors not visible to ward or other patients)**	☐	
**Access to outdoor visiting space**	☐	
**Comfortable soft furnishing in the family visit room (e.g. fabric sofa)**	☐	
**A room set-up that could be rearranged for different activities (e.g. playing on floor)**	☐	
**Activities such as books, games or toys in the family visit room**	☐	
**Access to technology accessories (e.g. plugs, WIFI)**	☐	
**Access to a TV**	☐	
**A colourful family room with artworks or decorations**	☐	
**Access to drinks or snacks during visits**	☐	
**A check-in with staff after the visit**	☐	

22. Please now rank those five elements from most important (one) to least important (five).

[**Online Only:** We will display the five elements selected by the participant in question 21]

Final Comments

23. Is there anything else you would like to share about what would help children during hospital visits?

WP2 Questionnaire Healthcare Professional

Section 1: About You

1. What is your age? (in years): __________

2. How would you describe your gender?
   - ☐ Male
   - ☐ Female
   - ☐ Non-binary
   - ☐ Prefer to self-describe: ________________
   - ☐ Prefer not to say

3. Are you a parent?
   - ☐ Yes
   - ☐ No
   - ☐ Prefer not to say

4. How would you describe your ethnicity?
   - ☐ White British
   - ☐ White other
   - ☐ Black/Black British
   - ☐ Asian/Asian British
   - ☐ Mixed/multiple ethnic groups
   - ☐ Other ethnic group: __________________
   - ☐ Prefer not to say

5. How long have you lived in the UK?
   - ☐ Less than 1 year
   - ☐ 1–5 years
   - ☐ More than 5 years
   - ☐ Prefer not to say

6. Current status in the UK (optional)

   People may have different experiences of living in the UK. Which of the following best describes your current situation?

   - ☐ Refugee (granted asylum or humanitarian protection)
   - ☐ Asylum seeker (waiting for a decision on an asylum claim)
   - ☐ Other visa or immigration status (e.g. student, work, family)
   - ☐ British citizen
   - ☐ Prefer not to say

7. Which county do you live in?:___________________________________

8. What is your job role?
   - ☐ Nurse
   - ☐ Healthcare professional
   - ☐ Psychologist
   - ☐ Psychiatrist
   - ☐ Occupational therapist
   - ☐ Research assistant
   - ☐ Other, please specify:______________________________
   - ☐ Prefer not to say

9. How long have you worked on adult psychiatric inpatient units?:

   ____

10. Are you currently working on a ward where family visits (including children) happen?
    - ☐ Yes
    - ☐ No

11. Have you personally supported a visit involving a child?
    - ☐ Yes
    - ☐ No

Section 2: Experience of Family Visits

12. Please rate the following statements:

	**Strongly Disagree**	**Disagree**	**Neither Agree to Disagree**	**Agree**	**Strongly Agree**
Our ward has a suitable space for family visits	☐	☐	☐	☐	☐
Children are supported appropriately during visits	☐	☐	☐	☐	☐
Parents are supported before and after visits	☐	☐	☐	☐	☐
I feel confident supporting family visits	☐	☐	☐	☐	☐
I understand the risks and safeguarding procedures	☐	☐	☐	☐	☐
I think family visits can benefit patients	☐	☐	☐	☐	☐
I am clear about family visit procedures / policies	☐	☐	☐	☐	☐

Section 3: Priorities for family visits

13. Below is a list of features which may contribute to how your ward facilitates child visits to parents who are patients. Please rate the extent to which those elements are present within your inpatient unit from one to five.

	Not Present **(1)**	Rarely Present **(2)**	Partially Present **(3)**	Frequently Present **(4)**	Fully Present/ Embedded **(5)**
Clear policy or guidance on family visits forward staff	☐	☐	☐	☐	☐
Staff training on supporting families	☐	☐	☐	☐	☐
A confirmation message of visit details sent to the family (e.g., by email or text) before visits	☐	☐	☐	☐	☐
Information for the family before the visit (e.g., what the hospital visiting room is like, rules about bringing items in)	☐	☐	☐	☐	☐

	Not Present **(1)**	Rarely Present **(2)**	Partially Present **(3)**	Frequently Present **(4)**	Fully Present/ Embedded **(5)**
Opportunity for the patient to plan the visit with staff beforehand	☐	☐	☐	☐	☐
Signs around the hospital to help the family find their way around	☐	☐	☐	☐	☐
A named staff member to support the family during visits	☐	☐	☐	☐	☐
Staff being available nearby during visits if needed	☐	☐	☐	☐	☐
Staff being present in the room during visits	☐	☐	☐	☐	☐
A dedicated room/space for family visits.	☐	☐	☐	☐	☐
Access to outdoor visiting space	☐	☐	☐	☐	☐
Visits being private (visitors not visible to ward or other patients)	☐	☐	☐	☐	☐
A brightly decorated family room with comfortable soft furnishing (e.g. fabric sofa)	☐	☐	☐	☐	☐
A room set-up that could be rearranged for different activities (e.g. playing on floor)	☐	☐	☐	☐	☐
Activities such as books, games or toys in the family visit room	☐	☐	☐	☐	☐
Access to technology accessories (e.g. plugs, WIFI) and a TV	☐	☐	☐	☐	☐
Access to drinks or snacks during visits	☐	☐	☐	☐	☐
A check-in with the patient after the visit	☐	☐	☐	☐	☐

14. Below is a list of features which may contribute to how your ward facilitates child visits to parents who are patients. Please select the FIVE elements which you feel are most important.

	**Select the five items which are most important**	**Rank the select items most important (one) to least important (five)**
Clear policy or guidance on family visits for ward staff	☐	
Staff training on supporting families	☐	
A confirmation message of visit details sent to the family (e.g., by email or text) before visits	☐	
Information for the family before the visit (e.g., what the hospital visiting room is like, rules about bringing items in)	☐	
Opportunity for the patient to plan the visit with staff beforehand	☐	
Signs around the hospital to help the family find their way around	☐	
A named staff member to support the family during visits	☐	
Staff being available nearby during visits if needed	☐	
Staff being present in the room during visits	☐	
A dedicated room/space for family visits.	☐	
Access to outdoor visiting space	☐	
Visits being private (visitors not visible to ward or other patients)	☐	
A brightly decorated family room with comfortable soft furnishing (e.g. fabric sofa)	☐	
A room set-up that could be rearranged for different activities (e.g. playing on floor)	☐	
Activities such as books, games or toys in the family visit room	☐	
Access to technology accessories (e.g. plugs, WIFI) and a TV	☐	
Access to drinks or snacks during visits	☐	
A check-in with the patient after the visit	☐	

15. Please now rank those five elements from most important (one) to least important (five).

[**Online Only:** We will display the five elements selected by the participant in question 14]

Final Question

16. Is there anything else you think would improve the experience of family visits for patients and their children?

____

____

____

____

____

____
